# Modular Data Acquisition System for Recording Activity and Electrical Stimulation of Brain Tissue Using Dedicated Electronics

**DOI:** 10.3390/s21134423

**Published:** 2021-06-28

**Authors:** Paweł Jurgielewicz, Tomasz Fiutowski, Ewa Kublik, Andrzej Skoczeń, Małgorzata Szypulska, Piotr Wiącek, Paweł Hottowy, Bartosz Mindur

**Affiliations:** 1 Faculty of Physics and Applied Computer Science, AGH University of Science and Technology, al. A. Mickiewicza 30, 30-059 Krakow, Poland; tomasz.fiutowski@agh.edu.pl (T.F.); skoczen@ftj.agh.edu.pl (A.S.); malgorzataszypulska89@gmail.com (M.S.); wiacek@agh.edu.pl (P.W.); hottowy@agh.edu.pl (P.H.); bartosz.mindur@agh.edu.pl (B.M.); 2 Nencki Institute of Experimental Biology, Polish Academy of Sciences, ul. Pasteura 3, 02-093 Warszawa, Poland; e.kublik@nencki.edu.pl

**Keywords:** data acquisition, microelectrodes, neurophysiology, electrical stimulation, neural interfaces, software engineering, ASIC

## Abstract

In this paper, we present a modular Data Acquisition (DAQ) system for simultaneous electrical stimulation and recording of brain activity. The DAQ system is designed to work with custom-designed Application Specific Integrated Circuit (ASIC) called Neurostim-3 and a variety of commercially available Multi-Electrode Arrays (MEAs). The system can control simultaneously up to 512 independent bidirectional i.e., input-output channels. We present in-depth insight into both hardware and software architectures and discuss relationships between cooperating parts of that system. The particular focus of this study was the exploration of efficient software design so that it could perform all its tasks in real-time using a standard Personal Computer (PC) without the need for data precomputation even for the most demanding experiment scenarios. Not only do we show bare performance metrics, but we also used this software to characterise signal processing capabilities of Neurostim-3 (e.g., gain linearity, transmission band) so that to obtain information on how well it can handle neural signals in real-world applications. The results indicate that each Neurostim-3 channel exhibits signal gain linearity in a wide range of input signal amplitudes. Moreover, their high-pass cut-off frequency gets close to 0.6 Hz making it suitable for recording both Local Field Potential (LFP) and spiking brain activity signals. Additionally, the current stimulation circuitry was checked in terms of the ability to reproduce complex patterns. Finally, we present data acquired using our system from the experiments on a living rat’s brain, which proved we obtained physiological data from non-stimulated and stimulated tissue. The presented results lead us to conclude that our hardware and software can work efficiently and effectively in tandem giving valuable insights into how information is being processed by the brain.

## 1. Introduction

Brain activity can be examined using various techniques, some of which measure electric signals directly reflecting neuronal activity and interactions. Noninvasive electrophysiological approaches dominate human research (Electroencephalography (EEG) [1] or Magnetoencephalography (MEG) [2]), while basic research, with animal models, mostly use electrodes and Multi-Electrode Arrays (MEAs) [3] implanted deep into studied structures. Each of these methods covers specific research scope regarding spatio-temporal resolution and the type of information that can be extracted from recorded signals. EEG and MEG give collective information about the activity of relatively large regions of the brain [4]. MEAs record neural signals of single cells or their populations.

The complementary experimental approach is to influence neuronal activity. Inhibition or stimulation of a particular neuronal population reveals its impact on brain functioning and animal’s behaviour. Classically, such an influence can be obtained by the use of neuropharmacological compound acting on receptor activating or inhibiting the neurons, or by the direct electrical stimulation [5]. Recently, a new method called optogenetics [6] was developed: neurons are forced to produce membrane proteins (opsins), which—upon delivery of a light impulse—lead to membrane depolarisation (i.e., activation of a neuron) or hyperpolarisation (i.e., its inhibition) [7]. Optogenetics became very popular because of the high specificity of neuron types in which the opsins can be expressed. However, electrical stimulation has certain advantages over optogenetics. Microelectrodes provide a two-way communication interface—the same probe can be used to record and stimulate the tissue with unprecedented spatial resolution. Single recording point on a silicon-based printed MEA can have a diameter below 10 μm, while optic fibres typically are 100 μm thick. MEAs can have hundreds of recording (and stimulating) points, while optogenetics uses single fibres or printed boards/probes with few micro Light Emitting Diodes (μLEDs). Furthermore, it is still not clear to what extent genetically modified, light-sensitive neurons preserve their physiological functions and exhibit the same information processing protocol as their non-modified counterparts [8,9]. On the other hand, delicately implanted, fine silicon probes induce minimal change in the neighbouring tissue.

The rapid development of MEAs’ fabrication techniques enables increased density and a smaller area of a single microelectrode for even more precise measurements [10,11,12]. However, these electrodes are passive elements, and their development has to be followed/paralleled by the development of adequate electronic devices, able to amplify and filter electric signals from all MEAs’ sites simultaneously [13,14]. This is not a trivial task because of the complexity of neural signals in frequency and amplitude domains. Fluctuation of membrane potentials are characterized by relatively slow sub-threshold changes and very rapid events due to supra-threshold stimulation [15] (*action potentials* or *spikes*). Action potential has an amplitude of around 100 mV when measured intracellularly. However, when recorded with extracellular MEAs spikes exhibit typical voltage amplitude from around 50 μV up to 1 mV and their waveforms correspond to a frequency of ∼1 kHz. Spikes are overlaid on a slower extracellular activity called Local Field Potential (LFP) [16], which reflects an average synaptic activity of a relatively large neuronal population. LFP frequencies span from 0.1 Hz (or even less) to hundreds of hertz and may have amplitudes of several millivolts. Moreover, the recordings are often contaminated by electric stimulation artefacts which can be an order of magnitude greater than LFPs themselves. Ideally, an active electronic device for electrophysiology should be able to process both types of signals introducing as little noise and distortion as possible in an amplitude and frequency domain and should not be saturated by artefacts. In so-called active probes, electronics is integrated with MEA on a single chip. Independent electronic systems are more universal as they are capable of cooperating with a number of commercially available MEA’s types. In order to take full advantage of MEAs experimental capabilities, such a device should also provide current or voltage sources. These would enable programmable stimulation with electrical pulses of arbitrary shape delivered through any of the MEA’s sites [17]. Finally, the connection with the other parts of the Data Acquisition (DAQ) system has to be realised with lightweight cabling or even wirelessly so that to ease their manipulation.

To meet these expectations, the device should be designed and fabricated as Application Specific Integrated Circuit (ASIC) with the functionality of output channels’ data multiplexing into a few signal lines. Usually, there are several such ASICs working in parallel in a DAQ system providing the access to hundreds of MEAs’ sites (also referred as *channels*). Their operation has to be synchronised so that to obtain all data samples at the same moment. The easiest way to achieve this is to supply each ASIC with a continuous stream of data (*Output Bitstream*) which is meant to control their real-time operation. Such *Output Bitstream* has to be created efficiently by dedicated DAQ software running on the Personal Computer (PC) communicating with the rest of the DAQ hardware. The experiment controlling software needs also to be able to:

- Recover samples of data recorded by each hardware channel from the multiplexed input.
- Provide experiment control mechanisms.
- Efficiently store sampled data as well as any other information that could be useful from any further data analysis perspective (e.g., markers of an experimental paradigm).
- Visualise data according to the experimental conditions and user’s preferences.

All of these tasks need to be performed reliably in real-time. Hence, the software must be well optimised and tested.

Here we present the DAQ system designed specifically to work with a custom-designed ASIC called Neurostim-3 [18] (its internal architecture was entirely arranged and optimised for the purposes of electrophysiological experiments by us) and commercially available MEAs in the in vivo experiments on anaesthetized animal (e.g., mouse, rat) brains. The Neurostim-3 ASIC provides a possibility to sample electric potentials of neurons and generate arbitrarily complex output current patterns on each of its channels independently and simultaneously. The inseparable part of the presented DAQ system is the dedicated i.e., built from the ground up software enabling efficient conducting of experiments even with thousands of generated pulses per second and real-time customisable data visualisation.

In this paper, we aim to:

- Present hardware architecture of the custom-designed electronics that can be used in electrophysiological experiments with a living brain.
- Show in-depth organisation of the DAQ software accentuating challenges and applied solutions.
- Discuss system performance regarding the quality of signal processing of the ASIC, software stability and resource management.
- Render preliminary results of data acquisition with living rat brains.
- Provide an outline of possible future experiments and software enhancements enabling more sophisticated paradigms and real-time data processing.

In order to fulfil these goals, this paper is structured as follows:

- Hardware architecture of the system and electric signal processing pipeline logic are presented in Section 2.1 and Section 2.2.
- DAQ software design including real-time data visualisation is described in Section 2.3.
- DAQ tests in terms of signal processing performance and biological experiment methodologies are outlined in Section 2.4 and Section 2.5 respectively.
- Section 3 is devoted to the presentation and discussion of the obtained results regarding software (Section 3.1) and Neurostim-3 ASIC basic signal processing capabilities (e.g., gain, noise, filter bandwidth; Section 3.2). Additionally, Section 3.3 provides examples from ongoing electrophysiological experiments performed using the presented system.
- Our findings and plans for further system enhancements are summarised in Section 4.

## 2. Materials and Methods

### 2.1. Architecture of the System

**Figure 1:**
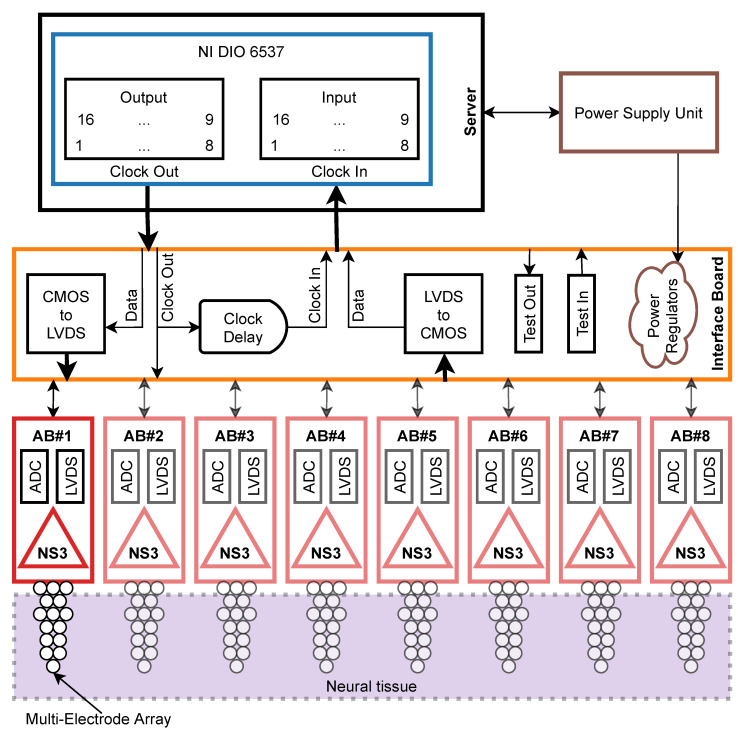
Hardware communication scheme of the presented system (object dimensions not to scale). NI DIO 6537 card (offering in total 16 output and 16 input digital data lines) resides in the Server and is connected with the Interface Board (IB) which manages signal and power distribution to/from up to eight ASIC Boards (ABs). Neurostim-3 (NS3) Application Specific Integrated Circuits (ASICs) are part of ABs and record electric potential changes on the Multi-Electrode Arrays (MEAs) dipped into neural tissue.

Our DAQ system consists of the two key components: (1) custom-designed hardware providing communication with up to eight Neurostim-3 ASICs able to acquire sampled neuronal signals and electrically stimulate neural tissue (2) driven by software platform responsible for control, management and visualisation of incoming data (Figure 1).

### 2.2. System Hardware

The hardware layer of the system provides physical means of electric signal generation, transmission, conversion, synchronisation and testing (Figure 1). These tasks are spread among several devices:

- Custom-designed elements:
  − ASIC Boards (ABs) housing Neurostim-3 ASICs and MEAs’ connectors.
  − Interface Board (IB) which distributes output signals and power among up to eight Neurostim-3 ASICs (currently available IB can handle two ASICs, but extension to the full system requires replication of a few IB elements only) and allows simple debugging procedures.
- Commercially available hardware supporting operation of IB and ABs:
  − National Instruments (Austin, USA) Digital Input/Output 6537 [19] measurement card installed inside the workstation *Server* and controlled by *NI-DAQmx Driver* [20,21]. The card is the bridge of data between IB and *Server*.
  − Rigol (Warszawa, Poland) DP1308A [22] Power Supply Unit (PSU) providing stable, low noise 5 V output with remote control possibility from the *Server* via General Purpose Interface Bus (GPIB) [23].

#### 2.2.1. Neurostim-3

**Figure 2:**
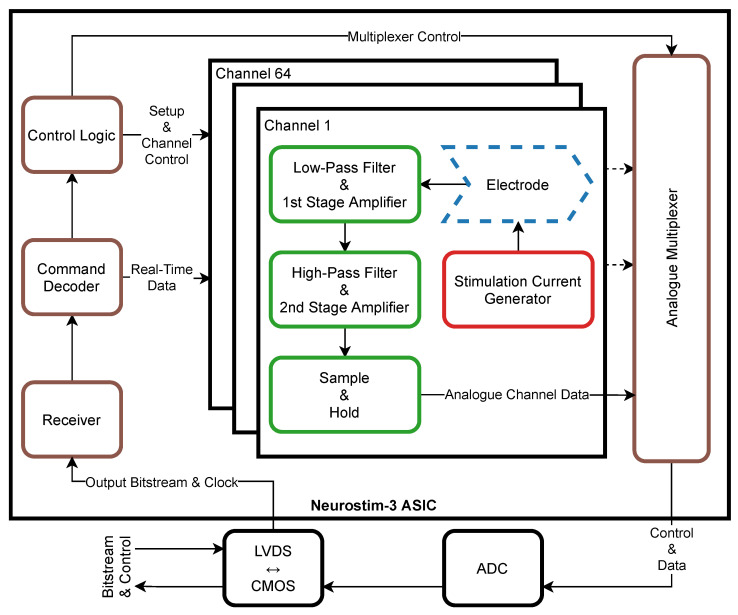
Data processing pipeline performed on the AB. *Output Bitstream* created by *Server* and distributed by IB comes to the Neurostim-3 ASIC. Before passing it to the ASIC, it is required to be converted back to a single-ended signal by Low-Voltage Differential Signalling (LVDS) to Complementary Metal–Oxide–Semiconductor (CMOS) converter. The device decodes the incoming stream of bits in order to prepare its states e.g., gain or cut-off frequencies and execute actions e.g., output current pulses or sample signals from connected MEA. Analogue samples from each channel are then multiplexed, converted to their digital form by Analogue-Digital Converter (ADC) and sent back to the IB as a differential signal.

The simplified block diagram of the Neurostim-3 ASIC is shown in Figure 2; the details of the chip design will be reported separately. The bitstream continuously developed by the *Server* workstation flown to each ASIC is logically a single line of data containing a mixture of setup commands and real-time information about electrical stimulation protocol for each of 64 ASIC channels. The bitstream is analysed first by the *Command Decoder* and depending on the command found, one of the actions below is scheduled (Figure 2):

- Set global ASIC parameter e.g., output current range, analogue multiplexer timing.
- Set per-channel parameter e.g., enable/disable electrical stimulation.
- Prepare each channel to drive current through an electrode and record signals based on the contents of the *Real-Time Data Command*.

In the latter case, signals at all active channels are sampled simultaneously according to the internally generated trigger. The occurrence of that trigger also results from *Real-Time Data Command* and, by default, all ASIC channels are sampled at 40 kHz frequency. Sampled signals are then processed by the two-stage amplifier and band-pass filter followed by sending over to the *Analogue Multiplexer* via *Sample & Hold* circuitry. Any further signal processing occurs outside the ASIC with devices installed on the AB.

#### 2.2.2. ASIC Board

**Figure 3:**
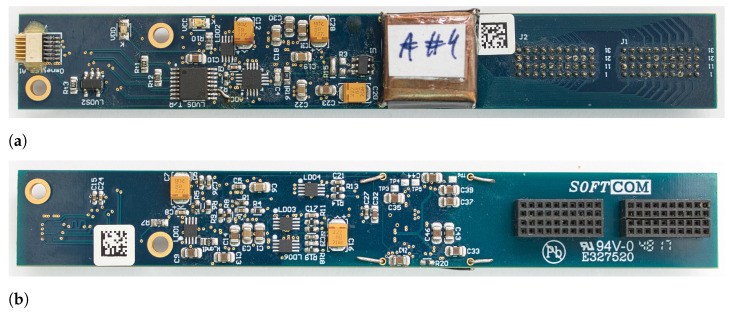
One of the possible configurations of AB with MEA connector (a) top view (b) bottom view. Neurostim-3 ASIC is not visible directly since it is enclosed inside the copper shielding (with the label A#4 indicating the particular copy of the device).

ABs are the components of the system the closest to an animal during the actual experiment. Each AB weighs 10 g and is 110 mm long and 15 mm wide (Figure 3). There are several key devices mounted there:

- Neurostim-3 ASIC under copper shield.
- Connector allowing usage of MEAs. Since there are many vendors of MEAs using different sockets [24,25], there are several versions of ABs to maintain compatibility with them. MEA becomes an interface between animal brain and Neurostim-3 ASIC. When dipped into the neural tissue, MEAs sample surrounding electric potential distribution changes due to the nearby cell population activity. They can also be used to output current driven by Neurostim-3 ASIC to artificially stimulate neurons.
- Analogue-Digital Converter (ADC) [26] converting multiplexed Neurostim-3 output data to its digital 12bit representation.
- Low-Voltage Differential Signalling (LVDS) [27] chip providing communication with the IB.

#### 2.2.3. Interface Board

**Figure 4:**
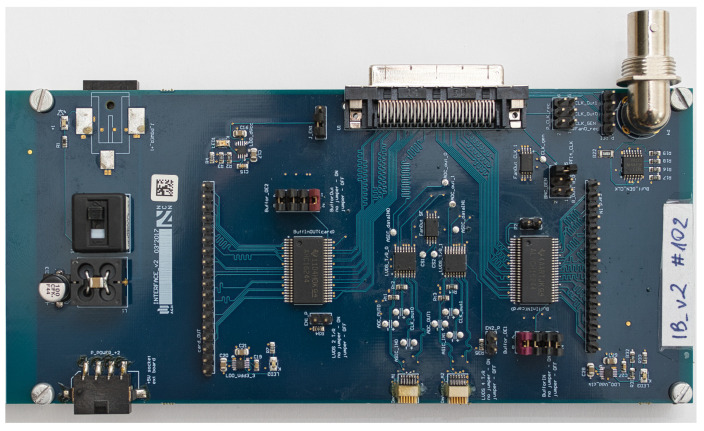
IB photograph. This particular version supports up to two AB and its dimensions are 15 cm × 7 cm.

Sequences of bits generated by the software are driven by NI DIO 6537 card to the IB (Figure 4). This card provides 16 output and 16 input digital Complementary Metal–Oxide–Semiconductor (CMOS) lines synchronised with clock signals. However, only the first eight output/input lines and clock source of 50 MHz are used by Neurostim-3 ASICs—others are reserved for special usage. Nevertheless, single-ended CMOS signalling provided by the NI DIO 6537 card is impractical when it comes to signal transmission over distances longer than 1 m due to the susceptibility to interference posing the threat of corrupted data. Moreover, Neurostim-3 ASIC does not incorporate any signal restoration protocol itself. That is why signals coming from the card are immediately transformed to differential form using CMOS to LVDS circuitry and only then sent together with the clock to appropriate ABs with Intan Technologies cabling [28] (one 0.9 m or 1.8 m cable maintaining two-way transmission).

Similarly to the output, received sampled differential signals from each AB need to be transformed from LVDS to CMOS before sending them to the card. There is no clock returning from ABs—it is rather looped on the IB from *Clock Out*, delayed and forwarded to the card as *Clock In*. The amount of the delay applied is adjusted so as to align valid input signals with the first edge of the clock. In other words, the duration of the delay results from the time difference between seeing the first bit of the *Output Bitstream* by IB and obtaining the first valid bit of sampled data as CMOS line.

Furthermore, IB exposes testing functionality and the possibility to control/be controlled by external devices. Card output signals (all 16 lines) may be examined with an oscilloscope using *Test Out* connectors. Analogically, *Test In* is used when aiming to send additional signals along with sampled data to the card. For example, one might want to drive external trigger signals using any of 9–16 input lines that can be used in software for custom event indications.

Various electronic devices used by our system (including Neurostim-3 ASIC itself) require stable power supply of 3.6V, 3.3V and 1.8V relative to the PSU ground. These are provided by Low-Dropout Regulators (LDOs) converting input 5 V from the PSU to desired levels on the IB. The voltage of 1.8V is used as a reference for the Neurostim-3 ASIC analogue readout. Thus, analogue signals reported here are presented relative to this level giving ±1.8V range.

### 2.3. Software Design

**Figure 5:**
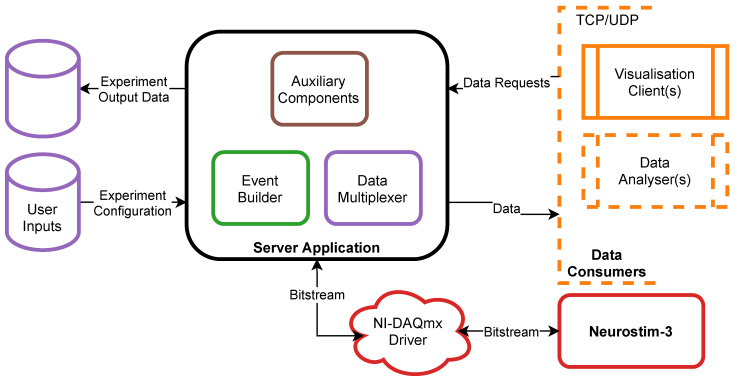
Schema of the core system architecture with the *Server Application* being its central part. *Server Application* is mainly responsible for (1) *Output Bitstream* creation based on the user-specified *Experiment Configuration*, (2) two-way communication with the NI DIO 6537 card which transfers data between the *Server* and Neurostim-3 ASICs, (3) extraction of meaningful per channel data from the *Input Bitstream*, (4) saving data onto permanent storage device (5) and further data distribution to compatible host applications via Transmission Control Protocol (TCP) or User Datagram Protocol (UDP).

Input/output communication with Neurostim-3 ASIC is carried out by custom-designed software platform that assures reliable connection with devices, data record onto non-volatile memory and its convenient real-time visualisation (Figure 5). Rather than being one monolithic system, it is a set of applications working in a client-server architecture. The main *Server Application* runs an experiment according to the user’s preferences and designed stimulation protocol, acquires input multiplexed data, and distributes it onto the hard drive and possibly to the one or more external *Data Consumers* (e.g., these may be *Visualisation Clients* or real-time *Data Analysers*). Data exchange between the *Server Application* and *Data Consumer* may be executed using either Transmission Control Protocol (TCP) point-to-point connection [29] or User Datagram Protocol (UDP) multicast transmission [30]. The first option is suitable when a consumer does not tolerate data losses at the price of a possible large network load, while the second uses a constant amount of resources regardless of the amount of the consumers but is prone to data losses. Such division of responsibilities not only simplifies the development of new *Data Consumers* but more importantly:

- Removes risks of data acquisition process failure due to any *Data Consumer* application error.
- Allows remote experiment control from many sites; moreover, one might want to run computationally extensive data analysis in real-time that is not possible to be performed using a single computer but might be split into smaller tasks operating on subsets of data on many machines, thus creating a computing cluster.

The software has been built entirely using *CPython* 3.8 programming language [31]. While, thanks to its active community, there are many tools and documentation [32] that speed up development, this language also has its challenges regarding efficient data processing. At the very core of the *CPython* interpreter, there is a mechanism called Global Interpreter Lock (GIL) [33] which prevents any two or more threads be executed at the same time in the given interpreter so that to prevent memory from unauthorised access. As a result, multithreaded applications will never take advantage of current multicore Central Processing Units (CPUs). To overcome this problem, the software uses multiprocessing to distribute data processing tasks among many *Python* interpreter instances. This, however, imposes another challenge being transfers of hundreds of megabytes of data between processes. By default, such data transfers are handled by *Synchronisation Manager*. This is an object controlling the process that mediates shared object exchange like queues, events, lists between cooperating processes [34]. *Synchronisation Manager* performs data serialisation whenever there is a request for communication between processes. This operation, however, is highly inefficient in terms of required CPU time, especially in the case of data transfers larger than 1 MB/s. To overcome this limitation, our approach is based on leveraging shared memory mechanism whenever there is a need to transfer large datasets (e.g., *Input/Output Bitstream*) between processes at the expense of a constant memory block allocation regardless of its actual usage.

**Figure 6:**
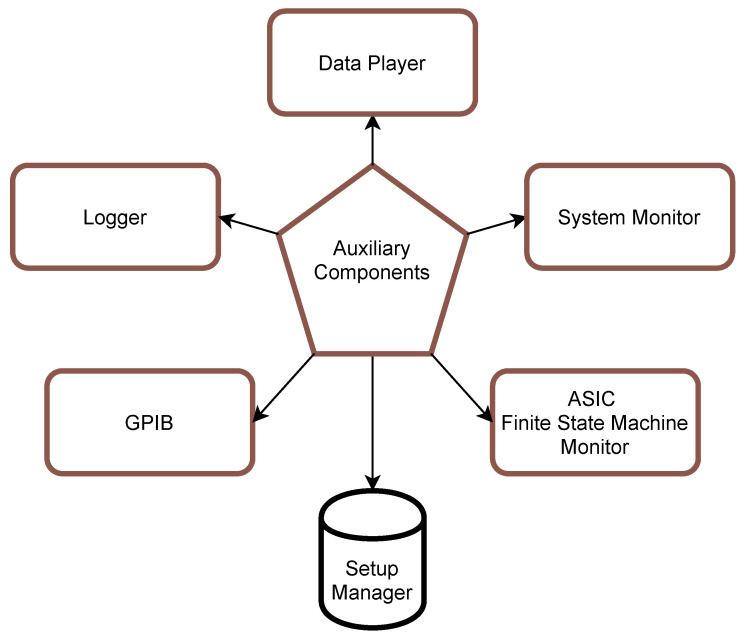
*Auxiliary Components* of the *Server Application*. Most of them: *Logger*, *System Monitor*, *ASIC Finite State Machine Monitor* are monitoring tools. *Data Player* allows replaying past experiments. *GPIB* offers a convenient way of communication with General Purpose Interface Bus (GPIB)-compliant devices. *Setup Manager* is a special database-like object keeping system critical constants in one place.

In addition to the main processing pipeline, the *Server Application* is also equipped with the set of *Auxiliary Components* that either provide better insight of its behaviour or simplify system operation (Figure 6):

- *GPIB* [23] provides remote control of the PSU.
- *Logger* gathers all information about the current state of each process and saves it for further investigation in simple text files.
- *Data Player* is meant to replay past experiment dataset files without the need for external tools. This way, one can pass the name of the dataset file and specific time range and then examine the data using *Visualisation Client*.
- *System Monitor* keeps track of all processes created by the system, monitors their resource utilisation and provides means of changing process priority and affinity for better performance.
- *ASIC control Finite State Machine Monitor* is a utility that in real-time tries to evaluate *Output Bitstream* contents to keep track of internal state registers of the ASICs. This tool comes in handy because no hardware mechanism would allow polling Neurostim-3 ASIC about its current state (*Input Bitstream* represents values sampled by each ASIC channel only).
- *Setup Manager* is a simple database-like file, keeping values of all critical parameters. Its instance is shared by all system processes, including *Data Consumers*.

#### 2.3.1. In-Depth Data Processing Pipeline Overview

To obtain valid *Input Bitstream* from the Neurostim-3 many consecutive processing steps need to be performed—these together form the data processing pipeline.

**Figure 7:**
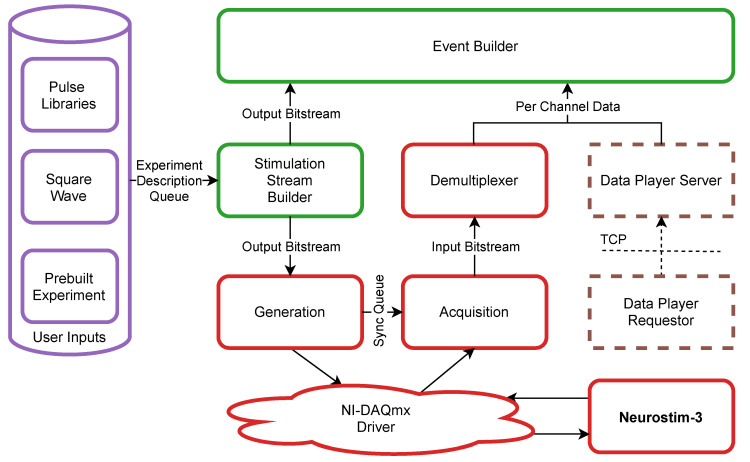
View of the bitstream generation and acquisition stages. The contents of the *Output Bitstream* depend on the required Neurostim-3 ASICs’ configuration and defined stimulation protocol — it is then sent over *NI-DAQmx Driver* to the Neurostim-3. *Acquisition* step actively awaits for the *Input Bitstream*, however, such data must be decoded by *Demultiplexer* in order to obtain *Per Channel Data*. Alternatively, it is possible to run the *Server Application* by replaying already archived datasets even without the presence of the Neurostim-3.

At the very beginning, the user must decide upon ASIC parameters that would determine Neurostim-3 operation e.g., output current range, signal gain, band-pass filter cut-off frequencies. However, most parameters are hidden from the user and sourced directly from the common *Setup Manager*. Furthermore, the user should provide information about the output current stimulation protocol. This can be achieved in several ways (Figure 7):

- *No protocol*—the device would operate in the read-only mode, which means it would only sample incoming signals with output current circuitry disabled.
- *Pulse Libraries*—user provides two *MATLAB* (MathWorks, Natick, USA) files [35], one of them keeps definitions of current pulse shape and amplitude, the second is a table telling when each channel should output given pulse.
- *Square Wave*—for testing purposes, it is beneficial to be able to output a continuous standard signal of arbitrary frequency to be applied on a subset of channels. Currently, both time and amplitude symmetrical square continuous wave pattern is supported.
- *Prebuilt Experiment*—this is a single file holding description of stimulation protocol and pulses used in JavaScript Object Notation (JSON) [36] format. Such a file is always created when the new experiment has been scheduled successfully and saved alongside the output dataset file.

Only if there is no problem detected with *User Inputs* the *Server Application* enqueues new experiment task onto *Experiment Description Queue*.

When the *Stimulation Stream Builder* process detects a new experiment task, it starts building *Output Bitstream* according to the provided Neurostim-3 parameters and stimulation protocol. Neurostim-3 setup is performed just once at the beginning of the experiment, and any further bitstream contains real-time data only. Since Neurostim-3 is designed to operate with 50 MHz clock whereas NI DIO 6537 has 16 output signal lines (lower eight are used by Neurostim-3 ASICs the other eight are reserved), there is a need to create 95.37 MB of data every second ($5\times 10^{7}$ samples, each of them is described by 16 bit value). Such a high fill rate is possible because pulses in the electrical stimulation protocol are typically sparsely distributed in time (up to several pulses per second). In addition to building regular bitstream, in all stimulation protocols but *Square Wave Stimulation Stream Builder* may introduce trigger signals on any higher *Output Bitstream* position whenever stimulation pulse is going to be output so that to indicate that one or more channels are currently injecting current. Such indication might be used by external hardware or wire-looped on the IB as a part of the *Input Bitstream*. Ready *Output Bitstream* chunks that correspond to the 0.5 s of operation are then sent with the queue to the *Generation* process.

*Generation* and *Acquisition* processes are the bridge between software and hardware through the communication with the *NI-DAQmx Driver*. This communication is maintained by the simple wrapper class allowing to call required low-level functions from the *NI-DAQmx Driver* Dynamic-Link Library (DLL) [37] directly, read internal state attributes (including error messages) and transmit actual data from/to our software. *Generation* and *Acquisition* processes are at the beginning responsible for proper setup of NI DIO 6537 card, and only then two-way continuous data transmission with Neurostim-3 ASICs occurs. *Generation* process enqueues to the driver subsequent *Output Bitstream* chunks without any change while *Acquisition* awaits multiplexed sampled data from each Neurostim-3 ASIC along with additional input signals (similarly to the output, lower eight input lines carry each Neurostim-3 ASIC multiplexed sampled data, while another eight inputs are treated as external trigger sources). This loop works as long as there are *Output Bitstream* chunks available for the *Generation* process. If that queue gets empty because of early user experiment termination, end of the experiment description without repeat procedure or excessive pulse injection during a long period of time (although queue is deep enough to keep four seconds of *Output Bitstream*) *Generation* process will start to repeat the last known *Output Bitstream* chunk in order to maintain communication with Neurostim-3 ASICs as long as there is no new chunk available. In that event, *Acquisition* is also receiving *Input Bitstream* from the *NI-DAQmx Driver* but needs the indication that these chunks should be dropped. This functionality is served by *Sync Queue* which is populated by *Generation* process with consecutive positive numbers normally, but when it schedules repeated chunk, it always puts −1 which indicates that *Acquisition* process should not enqueue such chunk for further processing.

If the *Input Bitstream* chunk passes the test with the next *Sync Queue* element, it is required to be demultiplexed in order to extract consecutive real values of sampled signal from each of the 512 channels and external trigger data. This task in case of ASIC channels requires multiple nested loops that are too slow in plain *Python* to be performed before new *Input Bitstream* chunk would be acquired (*Input Bitstream* chunk length also corresponds to 0.5 s acquisition). One way to overcome this bottleneck might be to create a standard compiled *C* language function library. Such solution, however, introduces external dependencies, so it was decided to use *Numba* just-in-time compiler [38] which translates *Python* source function to the optimised machine code for *Demultiplexer*. The output from the *Demultiplexer* is a two-dimensional (2-D) *NumPy* [39] array containing digital values sampled by all 512 channels during 0.5 s period (currently all channels are always setup to sample at 40 kHz rate) plus eight additional channels for binary trigger samples. Each value of this array is a 2 byte unsigned value so that to represent the dynamic range of the 12 bit ADC where 0 and 4095 correspond to a sampled signal of amplitude −1.8V and 1.8V respectively. In total, each *Per Channel Data* array takes as much as 19.84 MB that needs to be processed by further steps of the pipeline (the size of this array does not depend on the number of working ASICs—if one decides to use less than eight ASICs the vacant channels will always carry constant value data).

Alternatively, instead of running a new experiment, one might use the *Data Player* utility to replay already acquired data and use it for further processing steps e.g., data visualisation. *Server Application* runs *Data Player Server* process that constantly awaits data replay requests that can be sent remotely via TCP network connection. These tasks are issued by *Data Player Requestors* asking *Data Player Server* to rerun specific dataset file beginning and finishing at any point in time from that measurement. *Data Player Server* validates these requests and takes control over the *Per Channel Data* queue only if no experiment and no other replay is currently running.

**Figure 8:**
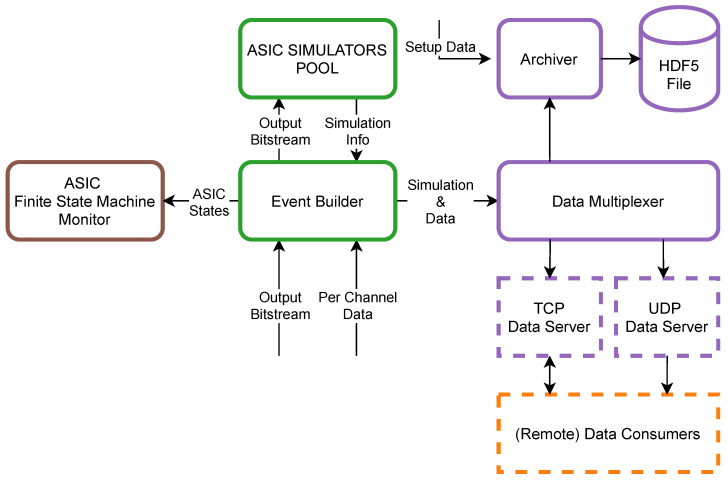
*Per Channel Data* distribution diagram. Acquired data and the corresponding *Output Bitstream* are put together by the *Event Builder* thanks to the *ASIC Simulators Pool* which programmatically simulate (1) state of each ASIC (2) and stimulation waveforms. *Per Channel Data* and simulated stimulation waveforms are then saved into the file and can be broadcasted by *TCP/UDP Data Servers* so that compatible host applications could make use of that data.

**Figure 9:**
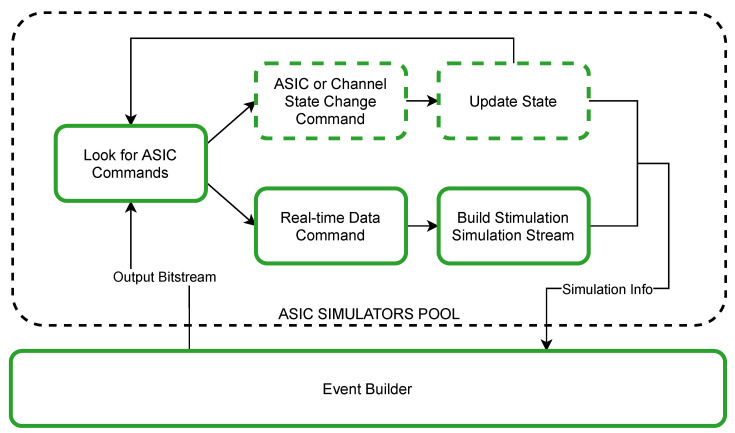
Simplified algorithm of the bitstream analyser used by the *ASIC Simulators Pool*. For each *Output Bitstream* chunk, the algorithm at the beginning looks for any command that is different from the *Real-time Data* and updates the state of the ASIC. If *Real-time Data Command* is found, it is assumed that there would be no state-related commands until the end of the current chunk.

The behaviour of each ASIC is simulated by a dedicated process. *Per Channel Data* with the copy of its corresponding *Output Bitstream* chunk are forwarded to the *Event Builder* process (Figure 8). It first distributes *Output Bitstream* to enabled *ASIC Simulators* in order to obtain *Simulation Info* which consists of update of each *ASIC Finite State Machine* and waveforms generated by current stimulation. The simplified algorithm of ASIC state simulation is presented in Figure 9.

When the simulator obtains a new *Output Bitstream* chunk, it starts its analysis. It is assumed that until the first *Real-Time Data Command* there might occur *ASIC* or *Channel State Change Commands* only and after the first *Real-Time Data Command* there will be a definition of stimulation protocol until the end of current *Output Bitstream* chunk. If *ASIC* or *Channel State Change Command* is detected, appropriate simulated ASIC state is updated whereas analysis of the chain of subsequent *Real-Time Data Commands* results with *Stimulation Simulation Stream* containing output current waveforms for all ASIC channels that are expected to be generated during that period. When *ASIC Simulator* finishes the processing of the current *Output Bitstream* chunk all its results are enqueued on *Simulation Info* queue and the process starts waiting for the next *Output Bitstream* chunk.

When *Event Builder* obtains *Simulation Info* from all active *ASIC Simulators* new *ASIC States* are forwarded to the *ASIC Finite State Machine Monitor* while generated waveforms are appended to the *Per Channel Data* array as *Simulation & Data* and forwarded to the *Data Multiplexer* process. Its only responsibility is to distribute obtained data to:

- *Archiver*—using Hierarchical Data Format (HDF5) file format [40] stores all *Simulation & Data* chunks with the system *Setup Data* on the permanent storage device as dataset file. These files can be used for offline data analysis by external tools or become an input for the *Data Player*. It is possible to enable gzip [41] data compression, which is especially useful when only a few Neurostim-3 ASICs are enabled to reduce the amount of data produced during the experiment.
- *TCP/UDP Data Servers*—allow external *Data Consumers* to connect to the stream of data. In the case of the TCP connection, *Data Consumer* may specify that it does not require data from all 512+8 channels, thus optimising network connection load.

#### 2.3.2. Visualisation Client

**Figure 10:**
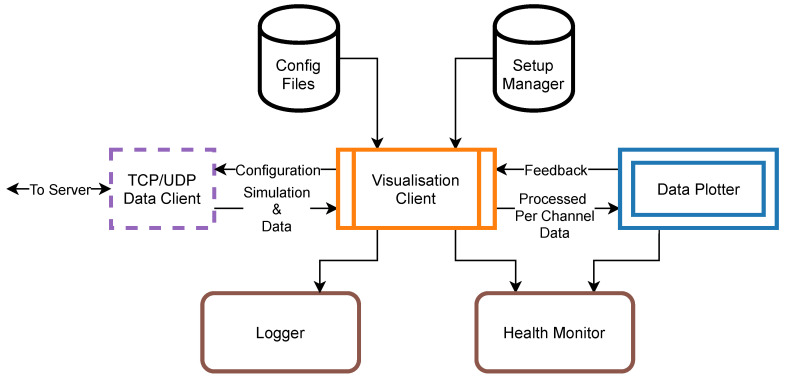
*Visualisation Client* organisation. When the *Server Application* is running, data can be collected by *TCP/UDP Data Client* then processed and plotted in accordance with the current visualisation configuration.

The most basic means of online control of the experiment is a visualisation of data produced by the hardware. In the system, this task is performed by the *Visualisation Client* which collects data from the working *Server Application* using either *TCP* or *UDP Data Client*, processes it according to the user’s preferences and draws waveforms onto the screen (Figure 10). Similarly to the *Server Application* this *Data Consumer* relies on the common *Setup Manager* database in order to provide proper working conditions. This tool also uses the *Logger* utility and *Health Monitor* as monitoring tools. The latter is a simplified version of the *System Monitor* and collects information about the amount of time that each pipeline step requires in order to process *Simulation & Data* waveforms.

**Figure 11:**
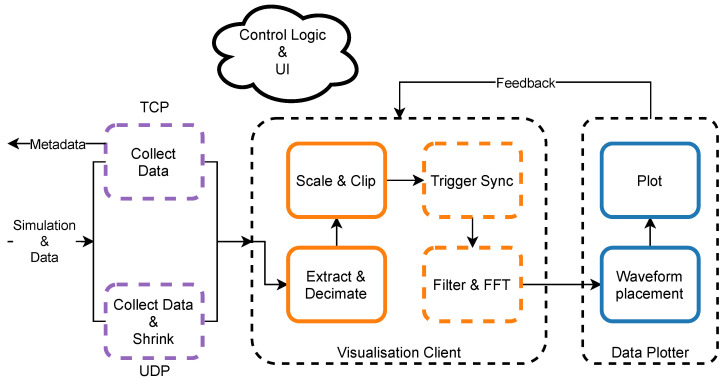
Visualisation data processing pipeline. In most cases, there is no need to plot data from all 512 channels. Therefore, only channels that can be presently visible are processed by subsequent stages.

Before starting the *Visualisation Client* user must decide upon which data transmission protocol would be used to receive data from the *Server Application* (Figure 11):

- When using TCP protocol *Visualisation Client* must provide the *Server Application* a list of channels that user is interested in. After a connection is established, the *TCP Data Server* sends this client only a part of the *Simulation & Data* corresponding to the requested channels, thus reducing the network load.
- UDP multicast transmission does not require any direct communication with the *Server Application*. *UDP Data Client* tries to collect as many packets as possible to build whole *Simulation & Data* chunk. All packets are indexed so that the client knows which packets were possibly dropped (if any). In the case of undelivered packets output, the data chunk would have holes. Only then data from the user required channels are extracted.

This way, the output of *TCP* and *UDP Data Clients* is consistent and contains data from the required channels only. Moreover, the list of channels may be changed at any time during *Visualisation Client* operation.

*Visualisation Client* was designed to present data waveforms from all 512 channels in real-time. However, each *Simulation & Data* chunk contains over $10^{7}$ individual values, which are not possible to render interactively. On the other hand, current 4K Ultra High Definition (4K UHD) displays are capable of drawing approximately 4000 points horizontally. That is why the second step after extraction of channel, trigger and stimulation data from the input array is data reduction by decimation. The software uses a minimum-maximum decimation algorithm in order to preserve information about rapid changes of sampled signal e.g., due to the neuron spiking activity. User can also adjust decimation factor interactively. However, the smaller the decimation factor and the more channels to process, the more CPU-demanding *Visualisation Client* is. Moving forward, channels’ data is scaled and clipped so that it contains data values from the specified voltage range only.

Having the trigger data, it is possible to align channels’ data with trigger event so that trigger pulse would be visible always in the same time position allowing to spot correlations between the two. User can pick from any of the eight external trigger sources—these can also be arbitrarily combined into one virtual trigger source. Moreover, it is possible to average responses around the trigger from the several consecutive trigger events, which is helpful when the user aims to filter out spontaneous signals not induced by the trigger source.

Furthermore, there is a simple Digital Signal Processing (DSP) module. Data, before sending it to the *Data Plotter* can be transformed by digital Butterworth filter [42] in case there is a special interest in the specific part of the signal spectrum during visualisation. One might use the low-pass filter with adjustable cut-off frequency and order when LFPs are of the greatest importance or high-pass configuration for a better view of neuron spiking activity. This way, thanks to the architecture of the DAQ the same input data can be split in order to visualise LFPs and spikes separately. Moreover, Fast Fourier Transform (FFT) [43] of channels’ data is possible which helps to track specific signal frequencies.

Finally, processed data, trigger and stimulation simulation information are packed together into the *Processed Per Channel Data* and consumed by the *Data Plotter* process. Its main task is to distribute data, trigger and stimulation simulation waveforms on the 2-D canvas according to the layout file specified by the user. The layout files describe positions where each channel waveform should be drawn and often reflect the physical layout of the probe microelectrodes used in the experiment. We chose *PyQtGraph* library [44] as the third party plotting tool since it is easy to use, versatile and proved to render data waveforms very fast within the specified 0.5 s time budget. *Data Plotter* also offers a possibility to pick only a subset of layout channels for visualisation and crosshair object that is used to examine signal levels of any plotted channel.

#### 2.3.3. Graphical User Interface

Both *Server Application* and *Visualisation Client* can be controlled by a user with sets of graphical controls allowing the change of numerous parameters according to the requirements of the experiment. Graphical User Interface (GUI) was designed using development tools distributed together with *PyQt5* library [45] in order to obtain a responsive layout of controls and portable design.

**Figure 12:**
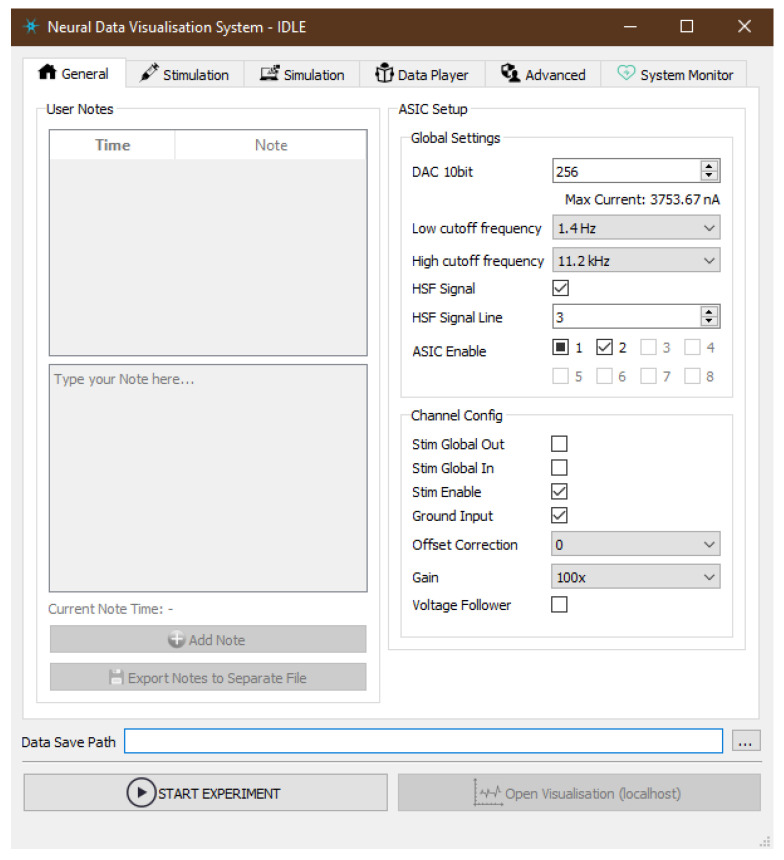
The main window of the *Server Application* allows setting up of experiment conditions including ASIC operation parameters and electric stimulation protocol. The interface is organised in tabs—each of them gives insight into the specific subsystem e.g., *Data Player* gives an opportunity to plan how to replay past experiment datasets while *Simulation* prints Finite State Machine (FSM) registers of enabled *ASIC Simulators* during data taking experiments with Neurostim-3 ASICs.

The interface of the *Server Application* reflects functionalities of the underlying subsystems and exposes the most useful parameters to be tuned by a user (Figure 12). For easier accessibility, GUI is thematically organised in the following tabs:

- *General*—exposes the ASIC parameters that would be applied in hardware at the beginning of the measurement. *Global Settings* define maximum output current value (*DAC 10bit*), hardware band-pass filter options and allow enabling individual ASICs. *Channel Config* is additional set of states applied at per-channel level in order to enable specific parts of its electronic circuitry like output current source (*Stim Enable*) or signal gain which can be set to seven discreet levels between 100 V/V and 500 V/V. Other visible parameters are useful when it comes to Neurostim-3 ASIC testing and debugging procedures. Although each ASIC can be configured independently of each other, currently we do not explore such a possibility.
- *Stimulation*—allows loading files describing output current stimulation protocol of an experiment.
- *Simulation*—when any of *ASIC Simulators* is enabled, one could examine here internal state of Finite State Machine (FSM) and choose how accurate in terms of the number of samples data simulation is.
- *Data Player*—configures dataset replay schedule.
- *Advanced*—enables power cycle procedure of the Neurostim-3 hardware right before the new experiment is scheduled. Moreover, here one can decide upon whether apply GZIP (GZIP) compression to saved data or not.
- *System Monitor*—presents summary as well as detailed information about hardware resources being used by DAQ.

**Figure 13:**
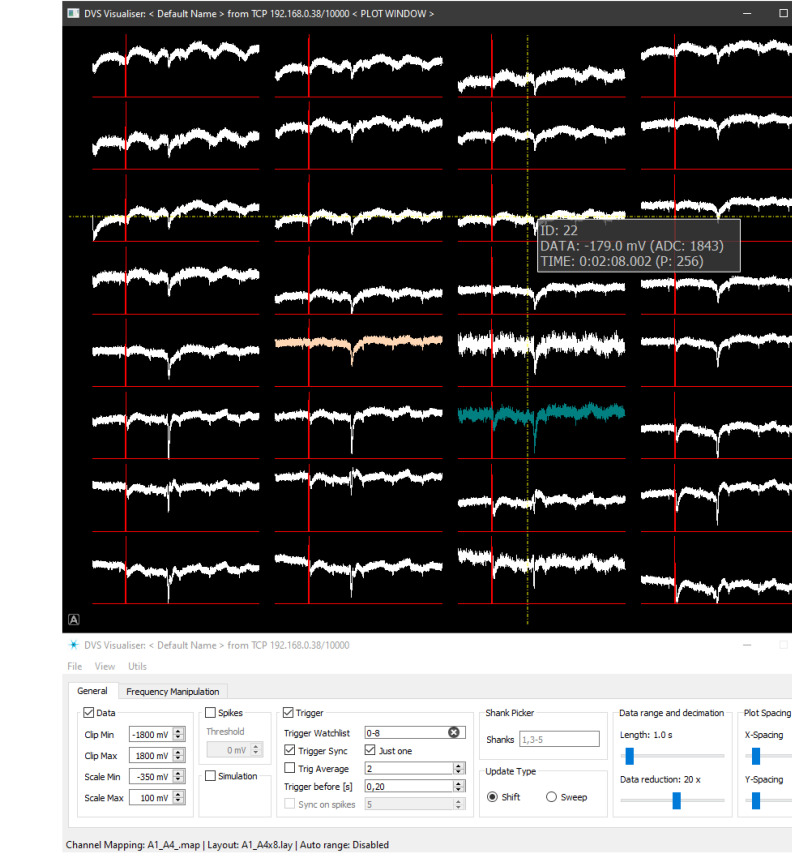
Graphical User Interface (GUI) of the *Visualisation Client*. The bottom window provides customisation of the visualisation parameters like input signal amplitude range, triggering, plot layout file and amount of data to present. The top window is the actual plot of data—in white, there are presented waveforms from the individual channels placed on the canvas according to the supplied layout file (some of them may be presented in different colours if they were marked by the user for better visibility); in red there is shown common trigger signal which can be chosen to align plot channel waveforms with the occurrence of the trigger.

*Visualisation Client* spawns two windows: one of them is the canvas where waveforms are being drawn, the second is the console with tunable parameters directly affecting visualisation data processing pipeline (Figure 13). The most crucial parameter for the entire visualisation is the mapping between MEA’s sites and ASIC channels resulting directly from the wiring between the probe and ASIC on the IB.

There is a possibility to draw at most the last 10 s of data. Waveforms may be scaled to present samples from the arbitrary input voltage range, aligned with trigger signals or examined using a precise data picker. User can highlight specific data channels and visualise data only from such subset, which is especially useful when ASIC is undergoing testing procedures. The visualisation configuration can be saved/loaded from a configuration file for convenience.

#### 2.3.4. Software Dependencies

Although our software highly relies on third-party solutions (refer to Table 1), there were many areas we had to cover ourselves, especially when it comes to *Output Bitstream* creation, *ASIC Simulation* and data visualisation pipeline. As a result, the production version of the code (excluding automatically generated GUI and third-party codes) consists of 77 files, 138 classes (including 33 *data classes* which serve simple role of data structures [46]) and over 13,500 lines of code (excluding empty lines and comments).

As of now, the minimum required version of *CPython* is 3.7. However, we are currently running our experiments in *CPython* 3.8.3 environment along with software packages mentioned in Table 1.

### 2.4. Neurostim-3 Testing Basis

Operational verification and test data quality analysis produced by Neurostim-3 ASIC was conducted using solely in-built capabilities of this device. This means that all signals, except for biological experiments, were generated by ASIC itself and recorded at the same time. To understand how this was achieved, a basic knowledge of Neurostim-3 ASIC capabilities and its communication protocol need to be presented briefly.

*Output Bitstream* produced by *Server Application* is in fact a continuous stream of commands alternating ASIC behaviour. The most frequently used command is the so-called *Real-Time Data Command* which schedules each active channel to output a specific amount of current and sample input signal simultaneously. This command is built of fixed-length sets of bits where each set alone describes the behaviour of each channel during that sampling period—in particular, the output current produced by Digital-Analogue Converters (DACs). Consecutive bits of each set affect states of e.g., built-in *DAC 7bit*, *DAC 4bit*, current polarity *p* and need to be adjusted by *Stimulation Stream Builder* in consecutive *Real-Time Data Commands* in order to obtain stimulation pulses of arbitrary shapes. These parameters, however, do not describe maximum achievable current amplitude, which is steered by ASIC global *DAC 10bit* register. This register has to be set with a command before any *Real-Time Data Command* occurs. Thus, the final temporary output current value can be calculated using the following formula:

(1) $$I_{o}\left(DAC,p\right):=\frac{\left[DAC\;10bit\right]\;\cdot \;\left[DAC\;7bit\right]\;\cdot \;\left[DAC\;4bit\right]}{\left(2^{10}-1\right)\left(2^{7}-1\right)\left(2^{4}-1\right)}\;\cdot \;15\mu A\;\cdot \;\left\{\begin{array}{cc} -1 & \mathrm{if}\;p=0\\ 1 & \mathrm{if}\;p=1 \end{array}\right..$$

The current produced by a channel might either be output to the MEA (which is the default behaviour during biological experiments) or connected to the channel input stage through the ∼5.5 kΩ resistor as if it was recorded from the electrode. The input signal undergoes then standard hardware processing pipeline consisting of band-pass filtering and amplifying stages. High-pass and low-pass filter cut-off frequencies can be configured using 5 bit registers. In the case of a high-pass filter, this should allow cut-off frequency adjustments between 0.6 and 7 Hz while the low-pass filter is expected to be tunable from 1 kHz to >20 kHz. Input signal amplification *g* might be set to any specified value g∈{100,150,200,250,300,400,500}V/V. This way, during presented test measurements, knowing stimulation current $I_{o}$ and gain *g* the expected signal amplitude $V_{\mathrm{ADC}}$ can be estimated as:

(2) $$V_{\mathrm{ADC}}(g,DAC,p):=g\;\cdot \;I_{o}(DAC;p)\;\cdot \;5.5\;k\Omega .$$

It can be shown that $V_{\mathrm{ADC}}$ may easily exceed the output dynamic range of the analogue readout −1.8 and 1.8 V. That is why in our tests, we adjusted current and gain in order not to exceed $V_{\mathrm{ADC}}=\pm 1$ V.

All the tests were conducted using the same AB and its internal testing features. It was placed in the closed metal grounded shielding box in order to cut down the possible electromagnetic background. The recorded datasets were analysed after the measurements.

#### 2.4.1. Software

The most important function of the software is to provide reliable communication with the Neurostim-3 ASIC. It had to be checked whether the designed communication protocol is maintained by the device during the test and real experiments. This was performed by directly looking at the visualised demultiplexed data and also printouts from *ASIC Finite State Machine Monitor* in multiple ASIC configuration scenarios. If there are any misinterpreted parameters by ASIC it will be most likely instantly reflected in the visualisation either as distorted waveforms or even no signal at all.

Since Neurostim-3 ASIC is designed so that to generate arbitrary output current patterns, it is also crucial to check the accuracy and performance of *Stimulation Stream Builder*. Pulse patterns should be observed on the exactly targeted channel and starting in the specific experiment time as designed in stimulation protocol. In order to check the performance of the *Stimulation Stream Builder*, the test based on *Pulse Libraries* was prepared to find out the maximum possible pulse rate persisting through a long period of time. The stimulation protocol assumed insertion of charge-balanced bi-phase pulses lasting for 250 μs. There were 20,000 pulses inserted in every second of the *Output Bitstream*— 1000 of pseudo-randomly distributed pulses on every of 20 non-adjacent Neurostim-3 channels (this results with 10,000 pulses per every *Output Bitstream* chunk, since each chunk describes 0.5 s operation of Neurostim-3 ASIC). All software pulse insertion optimisation mechanisms were disabled so that to be sure that insertions were performed one-by-one to simulate the worst-case scenario. *Stimulation Stream Builder* process performance was tested against four different process scheduling scenarios:

1. *Normal*—the process was running without process scheduling hints.
2. *Affinity*—the process was assigned to the 0th CPU core while the other processes of the DAQ could run freely on any but 0th CPU core.
3. *Priority*—priority of the *Stimulation Stream Builder* process was changed to *high*, while the rest of DAQ processes were running with *below normal* priority.
4. *Affinity + Priority*—two of the above combined.

The results shown in the following sections were obtained using *Server* workstation with Intel (Intel Corporation, Santa Clara, USA) Core i5-4590 4-core CPU without Hyper-Threading (HT) [54] and 16 GB Random Access Memory (RAM) working under Windows 7 operating system supervision (Microsoft Corporation, Redmond, USA). Our DAQ can also be run on a Linux system because there are no system-specific software dependencies and *NI-DAQmx Driver* is also available on this platform. Networking tests were performed by indirect connection of *Server* and *Client* computers via a switch using 1000 Mbit/s Network Interface Cards (NICs) (*Server*: Intel Ethernet Connection I217-LM [55], *Client*: Intel 82579LM Gigabit Ethernet PHY [56]).

#### 2.4.2. Inband Analogue Signal Response

Well-defined, uniform and linear channel response to the input signal defines reliability and quality of the data. It should be expected that the same stimulus would produce statistically the same recorded voltage, ideally across all ASIC channels. Moreover, knowing responses to well-defined stimuli with variable amplitudes, it is possible to determine channel gain linearity.

Analogue signal response characteristics were obtained based on the square pulse stimulation measurement with variable output pulse current amplitude. Neurostim-3 ASIC was set to transmit the broadest range of frequencies as possible, and measurements were conducted using signals of frequency close to the middle of the transmission band. One of the key assumptions was to measure all channels with all possible gain values so that to always operate within maximum recorded data sample value range around $V_{\mathrm{ADC}}=\pm 1\;V$. This could be achieved by scaling output current amplitude $I_{o}$ with changes of the expected gain value *g* i.e., the product of these parameters was kept constant and equal to 12,000  (Table 2).

**Figure 14:**
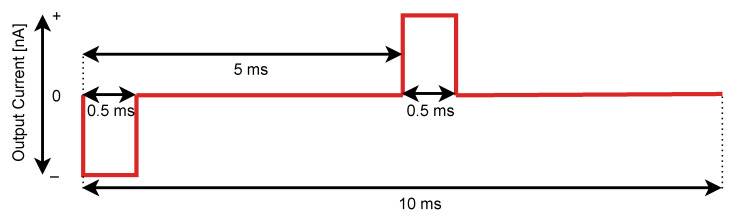
Single 10 ms period of the current pattern used to determine Neurostim-3 ASIC analogue signal response. Both pulses are of the same amplitude resulting from Digital-Analogue Converters (DACs) configuration but of different polarity.

Stimulation protocol for the fixed specified gain value *g* assumed scan of all ASIC channels with pulses of increasing amplitude. *Real-Time Data Commands* were built in a way that *DAC 4bit* was always set to its maximum which is 15 while *DAC 7bit* was adjusted from 2–127 with step equal to 5, giving 26 measurement series per each channel. Single stimulation period lasted 10 ms and consisted of two unipolar current pulses as presented in Figure 14. That sequence was repeated 1000 times for each *DAC 7bit* value. To eliminate dependencies between channels, only one channel was stimulated at a time.

Recorded datasets were used to obtain the mean input signal amplitude during each stimulation period for each channel. Average amplitudes were calculated based on the data samples obtained only during actual current output—samples from no stimulation subperiods acted as signal baselines. This procedure resulted in 26,000 values per each channel which were input to the plot of Neurostim-3 ASIC analogue signal response distribution.

#### 2.4.3. Gain

To analyse quantitatively spatial distribution of complex brain signals recorded at multiple electrodes in real-life experiment, the gain uniformity across ASIC recording channels and wide range of signal amplitudes is desired.

Measurements of gain rely on the data already gathered for finding ASIC analogue signal response. The only difference here is that all (1000) repetitions of each signal amplitude were averaged altogether to get the mean signal amplitude if the channel’s gain is set to *g* and stimulated with current $I_{o}$. This way, each channel was characterised by seven curves ${\bar{V}}_{ADC}\left(I_{o}\right)$—one per each specified gain setting. These curves were then approximated by a line in order to obtain actual values of gain $g_{fit}$ and signal offset $o_{fit}$ for each channel and expected gain setting. Additionally, to find whether channel signal gain depends on the signal being amplified or not, ${\bar{V}}_{ADC}\left(I_{o}\right)$ curves were divided by corresponding unamplified curves $V_{ADC_{u}}\left(I_{o}\right)=I_{o}\left(DAC\right)\;\cdot \;5.5\;k\Omega$—if channel gain is constant the result of this operation should also be constant irrespective of input signal amplitude.

#### 2.4.4. Noise

Since neural signals are of relatively low amplitudes i.e., from 50 μV to 10 mV, ASIC must amplify them introducing as little noise as possible. It is expected that for the fixed-gain value Power Spectral Density (PSD) of noise should decrease with an increase of frequency.

ASIC noise was measured based on the data taken with no stimulation protocol applied with all inputs grounded with 5.5 kΩ resistors. All channels were acquiring data at the same time for 3 h for each of the proposed gain values. The data was then extracted for each channel and gain settings in order to calculate periodograms showing PSD of noise in the frequency range from 0.1 Hz to 20 kHz. To do so, for each channel data and for the given gain, periodograms using Welch’s spectral density computation method [57] were obtained. Compared with regular periodogram calculation, this algorithm estimates signal PSDs in overlapping chunks of input data and only then they are averaged producing final channel signal PSD. This way, results are less noisy than those obtained traditionally. We used welch() function from the *SciPy* computing library [58] with 10 s single chunk length and 5 s data overlap on datasets not shorter than 10,000 s. All in all, there were at least 2000 individual periodograms used to obtain final noise PSD of each channel.

#### 2.4.5. Filtering

The ability to record non-distorted data in the broadest possible range of input signal frequencies is crucial for further experimental data analysis. It is especially important when LFP is being recorded when the lowest possible cut-off frequency of the high-pass filter is required.

In order to measure values of cut-off frequencies of each channel, *Stimulation Stream Builder* was creating continuous symmetric bipolar square wave pattern of frequency 0.1 Hz and output current amplitude 92 nA. There was a single channel being stimulated at a time for 5000 s resulting in 500 periods of data per channel. The data was obtained for high-pass and low-pass frequency registers set to 1 (∼0.6 Hz and ∼1 kHz, respectively) so that to be able to calculate the lower end of each filter of each ASIC channel. Additionally, for comparison, the same procedure was followed with different gain settings.

The recorded data from each channel in response to the exposition to the square wave stimulation pattern carries information about band-pass filter parameters. These, however, are not directly visible from the average time responses.

The band-pass filter implemented in each Neurostim-3 ASIC channel has transfer function given in Laplace transform domain as:

(3) $$K\left(s\right):=K_{0}\frac{sT_{H}}{\left(1+sT_{H}\right)\left(1+sT_{L}\right)},$$

where $T_{H}$ and $T_{L}$ are time constants of high-pass and low-pass branches of such filter, respectively. Test input signal is a bipolar square waveform of frequency $f_{w}=0.1\;\mathrm{Hz}$ and can be approximated by signed Heaviside step function ±H(t) which is expressed in Laplace transform domain as:

(4) $$\begin{array}{c} \gamma (t)=\pm H(t) \end{array}$$

(5) $$\begin{array}{c} L\left\{\gamma (t)\right\}=\pm \frac{1}{s} \end{array}$$

The ASIC filter response to the test stimuli is given by:

(6) $$Y\left(s\right):=\gamma \left(s\right)K\left(s\right)=\pm \frac{1}{s}\frac{sK_{0}T_{H}}{\left(1+sT_{H}\right)\left(1+sT_{L}\right)}=\pm \frac{K_{0}}{T_{L}}\;\cdot \;\frac{1}{\left(\frac{1}{T_{H}}+s\right)\left(\frac{1}{T_{L}}+s\right)}$$

which in the time domain is expressed as a linear combination of two exponents:

(7) $$\begin{array}{c} Y\left(t\right):=\pm \frac{K_{0}T_{H}e^{-\frac{t}{T_{L}}}}{T_{L}-T_{H}}\mp \frac{K_{0}T_{H}e^{-\frac{t}{T_{H}}}}{T_{L}-T_{H}} \end{array}$$

The derived Equation (7) describes time response to the input unit step of the first order active band-pass filter of arbitrary $T_{H}$ and $T_{L}$ time constants. These values can be extracted using standard curve fitting procedures to the averaged recorded data. In our case, the average absolute response to both half periods of the wave was calculated for each channel, so the fitting curve was simplified to the form:

(8) $$Y\left(t\right):=\frac{K_{0}T_{H}}{T_{L}-T_{H}}\left(e^{-\frac{t}{T_{L}}}-e^{-\frac{t}{T_{H}}}\right)$$

#### 2.4.6. Spike Pulse Stimulation

All previously described tests relied on the internally generated bi-phase square pulses. Neurostim-3 itself is capable of changing of output current in every sampling period allowing it to mimic much more complex signals. Therefore, in some sense, Neurostim-3 ASIC can be treated as if it was an arbitrary function generator with 64 independent channels. This can be invaluable when experiment conditions require advanced stimulation techniques e.g., stimulation artefact reduction [59]. That is why our DAQ was challenged to create and output complex signals resembling biological high frequency spiking activity. As previously, the same relationship between signal gain *g* and *DAC 10bit* was applied (Table 2). The output current pattern shape was obtained by high-pass filtering previously recorded neural signals with identified spiking activity. The source 2.5 ms spike pulse shape was approximated with 256 discrete levels by adjusting values of *DAC 7bit* and flipping current sign with polarity bit *p*. *DAC 4bit* this time was used to alter recorded spike pulse amplitude $V_{ADC}$ between 0.066 V and 1 V. The spike stimulation period was equal to 1 s, and each amplitude was applied 1000 times single channel at a time. For comparison, all stimulations were conducted twice—with minimum and maximum setting of the low-pass filter—in order to measure how low-pass filtration settings affect the recorded signals.

All recorded artificial spike pulses obtained for the same channel, gain, filter and *DAC 4bit* settings were averaged. Moreover, since this analysis aims to show how well Neurostim-3 ASIC can produce and transmit complex signals similar to biological, each of these curves was normalized to its maximum absolute value so that to enable direct comparisons of waveforms’ shapes with the source input waveform.

### 2.5. Biological Experiments

The system was tested in electrophysiological experiments studying the mechanisms of thalamo-cortical information processing. All experimental procedures followed the *2010/63/EU* directive [60] and adequate Polish regulations and were accepted by the 1st Warsaw Local Ethics Committee (protocol No 794/2018 accepted on 5th of December 2018). Animals were anaesthetized with *Uretan* (1.5 g/kg i.p.) and placed in a stereotaxic apparatus stabilizing the head in a reference position. The physiological state of an animal was monitored and maintained according to standard veterinary procedures. Small openings were drilled in a skull above the somatosensory cortex and thalamus in the right hemisphere to allow the implantation of electrodes. After completion of the recordings, rat received an overdose of pentobarbital (150 mg/kg i.p.) and was perfused transcardially with Phosphate Buffered Saline (PBS) followed by 10 % formalin in PBS. The brain was removed and cryoprotected in 30% sucrose solution. Coronal sections (50 μm thick) were cut on a freezing microtome and stained (for cytochrome oxidase) for microscopic verification of electrode positions.

**Figure 15:**
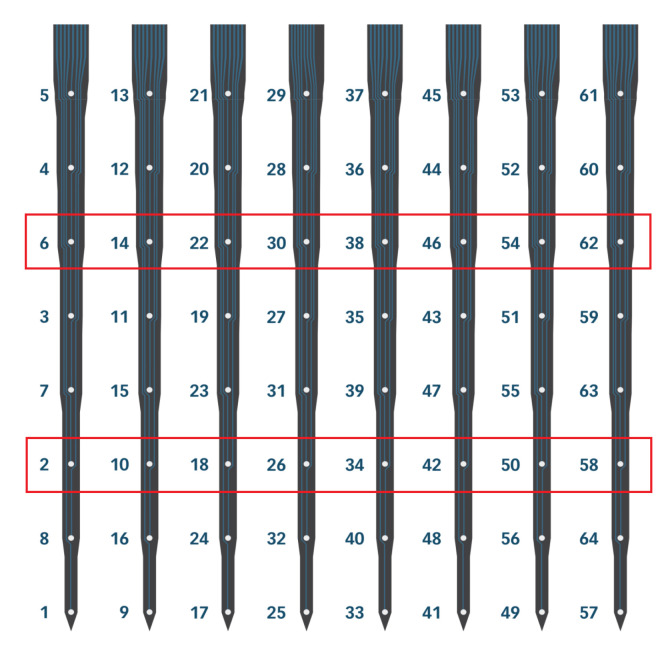
NeuroNexus *A8x8* MEA scheme used for conducting described experiments. Numbers next to each electrode tell the manufacturer’s electrode identifier. Red rectangles indicate MEA sites used for described experiments involving Neurostim-3 ASIC current stimulation capabilities.

Data examples presented here were obtained with two *A8x8* silicon MEAs (NeuroNexus Technologies, Inc., Ann Arbor, USA [61]) with a Ag/AgCl wire reference placed under the skin on the neck. Each probe has 64 iridium electrodes (recording and stimulating sites) of 13.3 μm diameter located on a regular 0.2 mm × 0.2 mm grid (eight points on eight probe shanks, Figure 15). Electrodes were electroplated with *NanoZ* (White Matter LLC, Seattle, WA, USA [62]) in gold-Polyethylene Glycol (PEG) solution [63] to reach impedance between 140 kΩ and 200 kΩ.

For the natural stimulation of the sensory system, rat’s left whiskers (i.e., sensory hairs on a snout) were attached to a piezoelectric slab, which was driven by 20 V square impulse to induce back and forth 0.1 mm movements. Electrodes were inserted into thalamic and cortical areas processing touch information to record a wave of activity evoked by such whisker stimulation. The *A8x8* probe records the signal from a 1.4 mm × 1.4 mm block of a tissue. Thus, after 100 repetitions of a stimulus, MEA in the rat’s barrel cortex was advanced by 1 mm deeper in order to probe the full depth of the cortex, and piezoelectric stimulation was applied again. Effectively, there was a 0.4 mm × 1.4 mm region that was sampled in both MEA positions.

Electrical stimulation can be used in order to activate neurons near the electrode. Here, we used the internal current stimulation capabilities of Neurostim-3 ASICs which also allow for simultaneous recording. Bipolar square-shaped pulses lasting 100 μs per phase of amplitudes between 0.7 μA and 5.6 μA were applied on electrodes residing in two MEA rows (marked with red rectangles in Figure 15) in both thalamus and barrel cortex. Electric pulses were generated every 2 s on both MEAs alternately in a way that stimulation period between new pulse generation on a given electrode was given by $T_{s}=2\times 16\times 2\;s=64\;s$. Pulses of each amplitude were applied 10 times on each electrode.

Natural and electrical stimulation experiments involved measurements of two distinct rats—one per experiment.

Neuronal signals (LFP and Evoked Potential (EP)) from the electrodes were recorded via two separate ABs simultaneously. Neurostim-3 ASICs were set up identically with maximum gain and signal frequency transmission band in the 0.6 Hz–10 kHz range. Whisker stimulation events were indicated by a digital signal acquired on one of the *Test In* channels on the IB so that to be able to precisely retrieve times when the piezoelectric stimulator was acting.

Recorded spontaneous LFPs and evoked responses were observed online in the *Visualisation Client* which helped choose the most active brain areas for offline analysis. Obtained datasets were analysed with external tools afterwards. Data from the measurements involving either whiskers or electrical brain stimulation from multiple stimuli were averaged in order to extract the mean tissue response for each recording site and stimulus parameters.

## 3. Results and Discussion

### 3.1. Software Metrics

**Figure 16:**
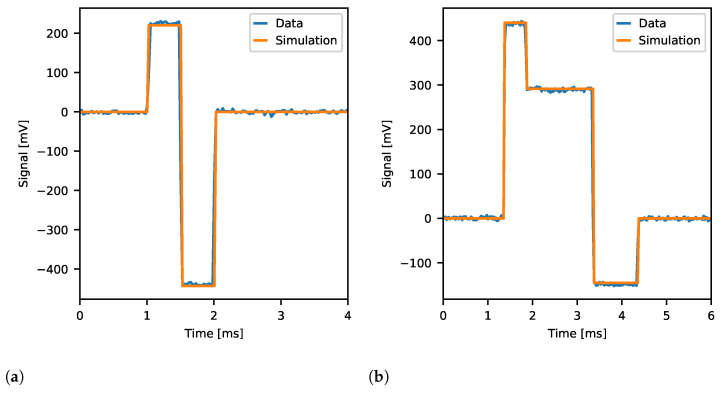
Current pulse stimulation waveforms of (**a**) diphase and (**b**) triphase pulses with their software simulation based on the real-time *Output Bitstream analysis*. The channel baseline level was subtracted from the data for better visibility and direct comparison.

Our tests show that *Server Application* is communicating with Neurostim-3 ASIC reliably. Internal states of ASIC are set according to the user requirements. Current pulse generation mechanisms work as expected with real pulses aligned with simulated stimulation (Figure 16). Simulated pulses occur on the right channels and at the same time as the pulses in acquired waveforms during measurements using Neurostim-3 ASIC internal testing procedures. Simulated stimulation events may be scaled both in time and amplitude per user request so that to make them more visible when data is being plotted by *Visualisation Client* during the actual experiment.

**Figure 17:**
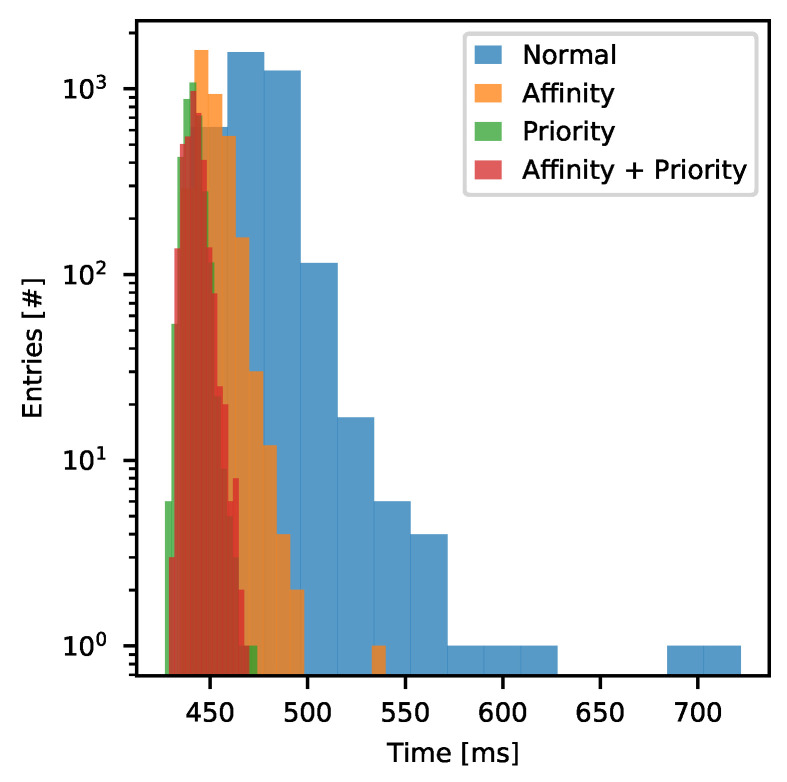
Performance of the *Stimulation Stream Builder* inserting 10,000 current pulses into 0.5 s *Output Bitstream* chunk. The distributions were obtained using different combinations of system processes scheduling options. *Normal* data was gathered when the operating system was given no additional preferences about running Neurostim-3 Data Acquisition (DAQ) processes. *Affinity* case reflects measurement when *Stimulation Stream Builder* process was assigned to the specific Central Processing Unit (CPU) core while the other DAQ processes were prohibited from running on that core. *Priority* data was taken with *Stimulation Stream Builder* process with assigned *high* priority, while the rest of them were set up to run *below normal* priority. *Affinity + Priority* is the combination of two of them. All stress tests passed without failure due to the *Output Bitstream* buffering.

The data processing pipeline of the *Server Application* is split between a dozen of cooperating processes and requires not less than 5 GB of RAM where 1.8 GB is always reserved by shared memory queues and *NI-DAQmx Driver* input/output buffers. The exact number of processes and memory usage depends on whether just one or both *Data Servers* are running and how many *ASIC Simulators* are in operation. Typically, running only *Server Application* with all *ASIC Simulators* disabled overall CPU utilization does not exceed 25%. Usually, the most CPU-intensive process is *Acquisition* with *Demultiplexer* which consumes up to 30% of the single core time. Since our *Demultiplexer* is implemented directly as machine code, thanks to the conversion provided by *Numba*, the time required to convert *Input Bitstream* chunk to *Per Channel Data* takes ∼74 ms. This result may be further improved because *Numba* can split computations between threads independent of the *Python* process. If using this feature, we can obtain ∼49%, ∼63% and ∼70% computation time reduction when this task is split between two, three and four threads respectively. It is worth mentioning that the computational complexity of *Acquisition* and *Demultiplexer* barely depends on the *Input Bitstream* contents. Thus, *Stimulation Stream Builder* may be even more demanding when the experiment description requires intensive stimulation. This was checked during the stress test of the *Stimulation Stream Builder*. Each test lasted for 30 min which means that *Stimulation Stream Builder* had to prepare 168 GB of data each time. The time of *Output Bitstream* chunk creation was measured as well as the mean CPU core utilisation by the *Stimulation Stream Builder* process. The distribution of *Output Bitstream* chunk preparation time for the discussed process scheduling cases is presented in Figure 17.

In all discussed cases, the software was able to keep up with the stimulation rate. However, no scheduling hints resulted in the poorest performance (Table 3). In that scenario, there were 3% of chunks that took longer to be built than the available single chunk time budget. Usually, this is not a problem because *Output Bitstream* chunks are buffered in the queue deep enough to store 4 s of data, so infrequent single performance drops can not affect the experiment. The performance of the *Stimulation Stream Builder* becomes much better when the process is pinned to the exact CPU core and is the best when processes’ priorities are adjusted. The latter case results with average 441 ms per chunk with very low time spread (σ = 4.2 ms) compared with *Normal* case with 7.4% worse mean time per *Output Bitstream* chunk and higher CPU core utilization. Combination of *Affinity* and *Priority* settings did not exhibit any visible improvement over *Priority* test case. All in all, we have confirmed that our DAQ software can sustain highly demanding experiment scenarios with up to 20,000 continuous pulse insertions per second. That performance metric can be even better by process scheduling adjustments or running DAQ on PC equipped with more powerful CPU in terms of single-core computation efficiency.

Since connection with *Data Consumers* is performed via a network, *Data Server* must be able to provide sufficient throughput in order to prevent data glitches and *Data Consumer* starvation. This was checked by sending a full stream of data consisting of data from all 512 channels, including *Stimulation Simulation* plus eight trigger sources. In such a case there is a requirement to maintain stable transfer of 48 MB/s (384 Mbit/s) per *Data Consumer*. We connected two computers indirectly via a switch using TCP protocol. One of them was running *Server Application* with a simple read-only test experiment, while the other was running one or more *Visualisation Clients* requiring full data input. When a single such a consumer is connected to the *TCP Data Server*, the system achieves 830 Mbit/s throughput, while running two such consumers increases this value up to 970 Mbit/s. The latter value is close to the maximum achievable using 1000 Mbit/s NIC and is sufficient to feed many *Data Consumers* that do not require all system channels information (that is the case during most experiments when single *Visualisation Client* usually plots information from not more than 128+8 channels at once). However, if presented transfers are not sufficient, one might switch to using *UDP Data Server/Clients* working in multicast mode if possible packet losses are not a problem or upgrade NIC and other network components to a higher standard like 2.5/5/10 Gbit/s.

The performance of the *Visualisation Client* depends heavily on a number of factors like the number of channels to process, decimation, length of data to plot, optional filtering, data transformations and even accuracy of the *Stimulation Simulation* data. Our tests indicate, however, that single *Visualisation Client* can interactively plot 0.5 s of 20× decimated data from all channels at once with filtering disabled. The filtering enabled in *Data Plotter* is a bottleneck of the data processing pipeline. If such performance is not enough, one might split the visualisation task between multiple clients processing subsets of all 512 channels.

### 3.2. Neurostim-3 Basic Characteristics

This section aims to present most of the DAQ capabilities in the form of the ASIC tests procedures. The presented test and procedures are normally applied when behavioural and functional validation of the ASIC design is performed.

#### 3.2.1. Inband Analogue Signal Response

**Figure 18:**
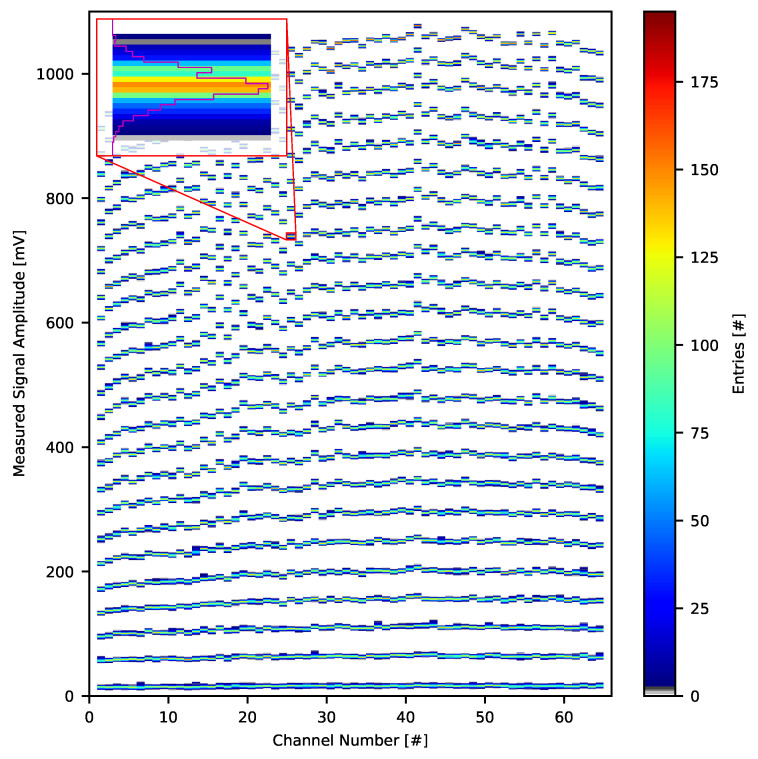
Measured signal amplitude distribution for gain setting g=250 and stimulation output current from 11 nA to 649 nA with a step of 28 nA. Each amplitude was recorded 1000 times for all of 64 Neurostim-3 ASIC channels.

The analogue signal response plots of all 64 Neurostim-3 ASIC channels for input current amplitude from 11 nA to 649 nA with a step of 28 nA and signal gain parameter set to g=250 are presented in Figure 18 (distributions for current amplitudes exceeding 650 nA are not shown). The distribution of the signal response is colour-coded, and its typical width is 10 mV. However, for current amplitudes exceeding 600 nA (measured signal amplitude ∼825 mV) distributions, become narrower due to the amplifier saturation. Moreover, an edge effect of channel gain non-uniformity can be observed, as extreme channels are showing lower gain than their centre counterparts.

#### 3.2.2. Gain

**Figure 19:**
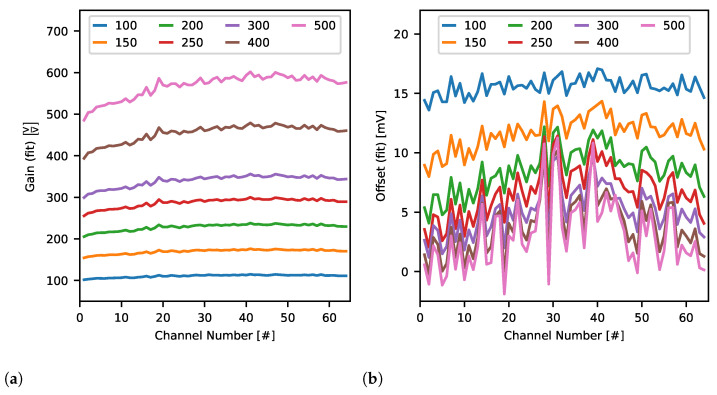
Measurement of channel (**a**) gain and (**b**) offset based on the relationships between stimulation output current amplitude and recorded signals’ amplitude.

The average response to each stimulation current was used to obtain a real per-channel gain and signal baseline values. Standard linear fit approximation was employed to evaluate the parameters. The gain values vary between channels being the lowest for side ones and the highest around channel 50 (Figure 19a). The overall shape of these curves does not depend on the gain. The shapes scale well with the gain values. Although such behaviour is undesired, it indicates proper operation of the Neurostim-3 ASIC. The most welcomed behaviour is to have very uniform gain values across all the ASIC channels. The offsets vary with gain (Figure 19b) and decrease for the higher ones. In all measured cases, the offsets have a similar shape as for the gains i.e., they are slightly lower for side channels.

**Figure 20:**
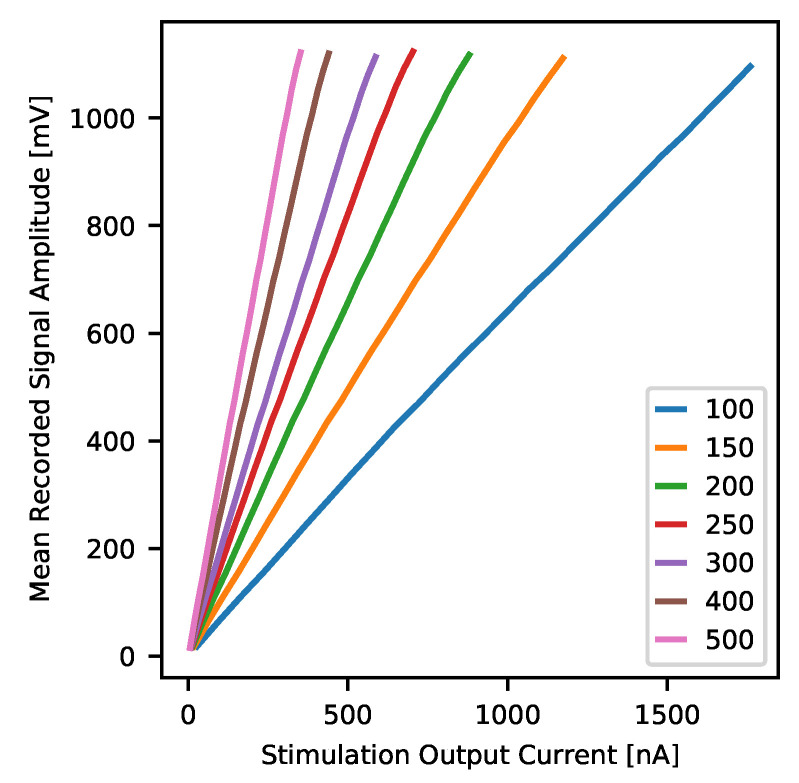
Sampled signal amplitude of the test channel resulting from stimulation output current for different gain settings.

For the randomly selected channel, we present the *test channel* average response in details. Obtained signals versus current dependencies are linear regardless of the gain setting in a broad range of recorded amplitudes (Figure 20).

**Figure 21:**
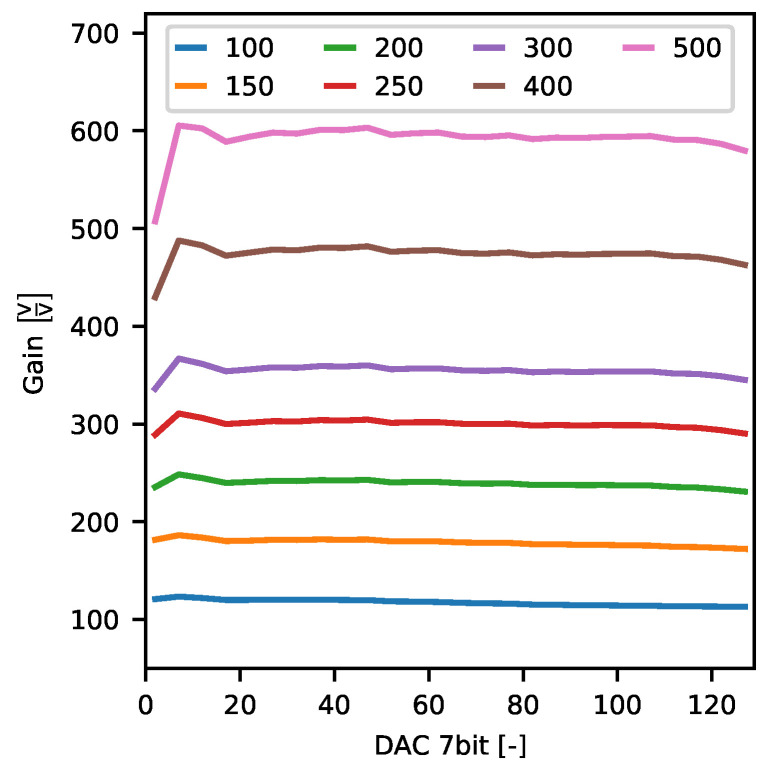
Measured gain of the test channel as a function of *DAC 7bit* value controlling the amplitude of the looped stimulation output current for different gain settings.

Finally, *test channel* gain stability as a function of *DAC 7bit* steering stimulation current amplitude is shown in Figure 21. Obtained curves are almost constant except for the extreme stimulation output currents. In that perspective, gain non-linearity is clearly visible for *DAC 7bit* values below 20 and is more apparent for higher gains. This is the result of the fact that gain settings and value of the global *DAC 10bit* register are balanced in our measurements (Table 2). Consequently, assuming the same input noise level, the effective Signal-To-Noise Ratio (SNR) is worse for higher gains and influences the results.

#### 3.2.3. Noise

**Figure 22:**
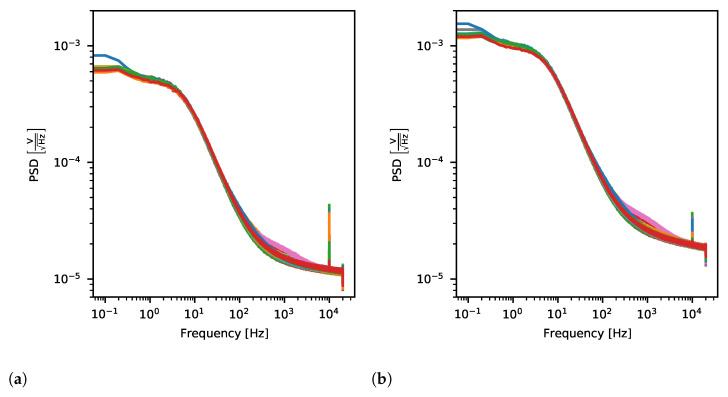
Power Spectral Density for all Neurostim-3 ASIC channels and gain (**a**) *g* = 250 (**b**) *g* = 500.

The output noise is uniform across all ASIC channels and increases with the applied gain (Figure 22). As expected, noise PSD is smaller for higher frequencies where the pink noise characteristic is affected by the band-pass filter. However, unexpected peaks at exactly 10 kHz are visible for almost all channels. Due to the fact that they are present for the quarter of channel sampling frequency, we suspect these peaks arise from the digital part of the system, outside Neurostim-3 ASIC itself. Still, the noise amplitude at 10 kHz does not exceed 20 μV RMS, which compared with amplified spikes is two orders of magnitude less and does not affect the quality of the acquired data.

#### 3.2.4. Filtering

Signal transmission properties are the most crucial when LFP data are recorded.

**Figure 23:**
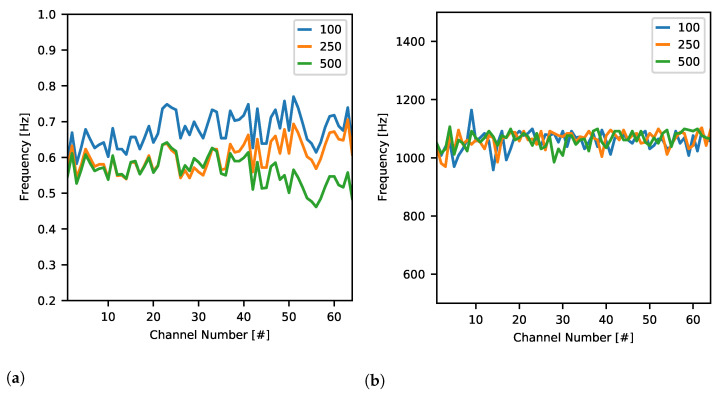
Minimum (**a**) high-pass (**b**) low-pass filter cut-off frequency obtained for the lowest possible band-pass filter settings and three different gains.

Figure 23a presents the distribution of channel cut-off frequencies of their high-pass filters with different gain settings applied. Although the curves are similar to each other, they are rather combinations of the gain-invariant and gain-dependent characteristics altering the final curves. One can note that for the lowest gain setting, the minimum cut-off frequency is around 0.7Hz while for the two higher presented gains, it drops to 0.6 Hz.

On the other hand, the low-pass filter is tunable from as low as ∼1050 Hz and is not affected by gain or channel number (Figure 23b). The visible spread of the cut-off frequency rather results from the ASIC production mismatch of individual components between ASIC channels.

The maximum cut-off frequency was also checked by setting the low-pass register to the maximum value (data not shown). We can only conclude that in these conditions cut-off frequency of each channel is not less than 14 kHz since that measurement was in this case limited by a data sampling frequency of 40 kHz.

#### 3.2.5. Spike Pulse Stimulation

**Figure 24:**
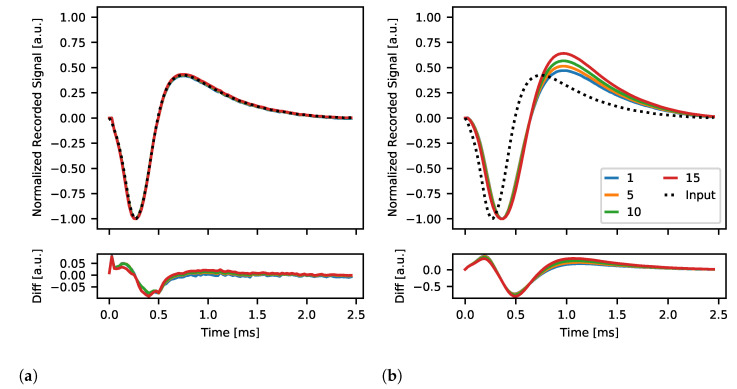
Normalized readout response of the test channel for artificial spike pattern stimulation at gain = 250 and different amplitudes controlled by DAC 4bit value with (**a**) maximum (**b**) minimum setting of cut-off frequency of the low-pass filter. Bottom plots show differences between obtained normalized signals and reference spike.

The ability of Neurostim-3 ASIC to produce and record complex signals is presented in Figure 24. The reference signal (in dots) represents the typical spiking activity curve which is to be mimic by the stimulation circuit of the Neurostim-3 ASIC in this test. The shape of the reference signal is compared with generated output current patterns of different amplitudes controlled by *DAC 4bit* and internally sampled by the *test channel*. There is very little difference between reference and recorded signals when the measurement was conducted for the broadest filter band (Figure 24a), confirming that Neurostim-3 ASIC is capable of generating arbitrary current patterns. On the other hand, when ASIC’s low-pass filters were set to the lowest value (that is around 1050 Hz as shown in Figure 23b), recorded signals were delayed and distorted which can be spotted during the positive phase of the signal (Figure 24b).

Presented results of arbitrary signal stimulation capabilities of the Neurostim-3 ASIC indicate that one can plan and perform any kind of stimulation patterns required for the actual biological experiments.

### 3.3. Biological Experiments

#### 3.3.1. Spontaneous and Whisker Evoked Activity

**Figure 25:**
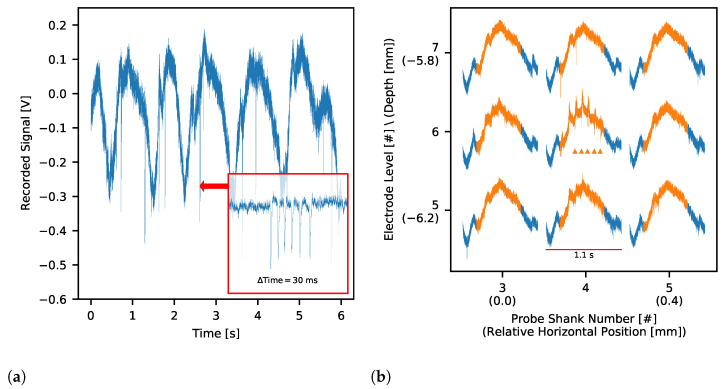
Wideband electrophysiological signals recorded with Neurostim-3 ASIC from NeuroNexus MEA placed in the thalamus of anaesthetized rats. Examples show Local Field Potential (LFP) typical for deep sleep. (**a**) Data from one channel showing large amplitude, slow waves and clear single unit bursting spiking activity. (**b**) Nine neighbouring channels with a slow wave and a sleep spindle–note ~12 Hz oscillations in LFP (highlighted parts of waveforms) with bursts of multi-unit spiking activity (arrowhead markers). Traces are drawn without gain correction of potential values.

The DAQ system was successfully used to record neuronal activity from two 64-channel MEA probes placed in the somatosensory thalamus and barrel cortex of anaesthetised rats. Recorded wide-band signal was composed of LFP with clear action potentials visible on multiple channels. Examples in Figure 25 show sweeps of spontaneous (without any stimulation applied) LFP with slow waves typical for a deep sleep. Figure 25a presents also action potentials (see zoomed window with burst of spikes lasting 10 ms) detected with a very good SNR. In the other part of that MEA (Figure 25b) we observe sleep spindles—short-lasting oscillations generated in a thalamo-cortical loop that are also characteristic for a sleeping brain [64]. This sleep spindle event (in orange on all sites) is prevalent on the central electrode where we see small LFP waves (∼12 Hz) in conjunction with spiking activity.

**Figure 26:**
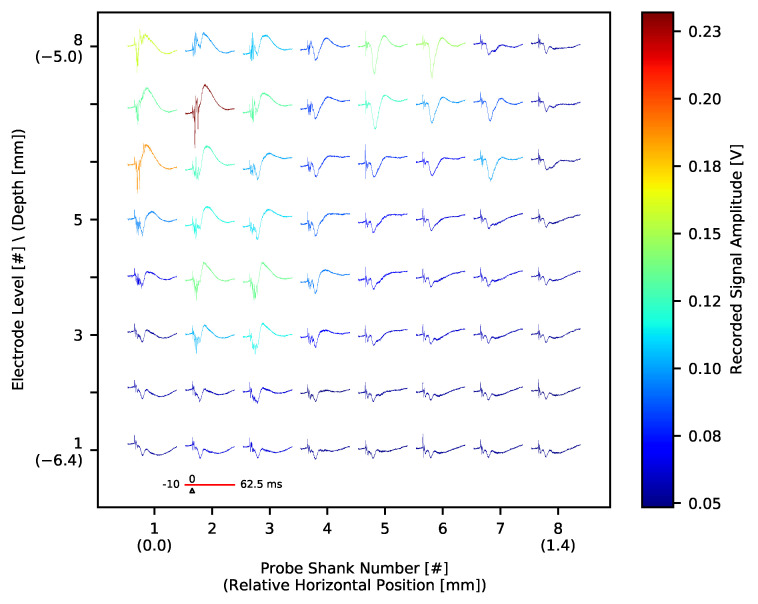
Average (n=100) evoked potential responses to whisker stimulation calculated for all MEA sites placed in a rat somatosensory thalamus. The largest amplitude and shortest latency of responses indicate that the electrodes in the top-left corner of the drawing were located in the region of the representation of large whiskers. In other channels, we record volume conducted signals of diminishing amplitude. Each average response is 62.5 ms long with 10 ms pre-trigger LFP and its amplitude is colour-coded (note that gain correction was not applied).

**Figure 27:**
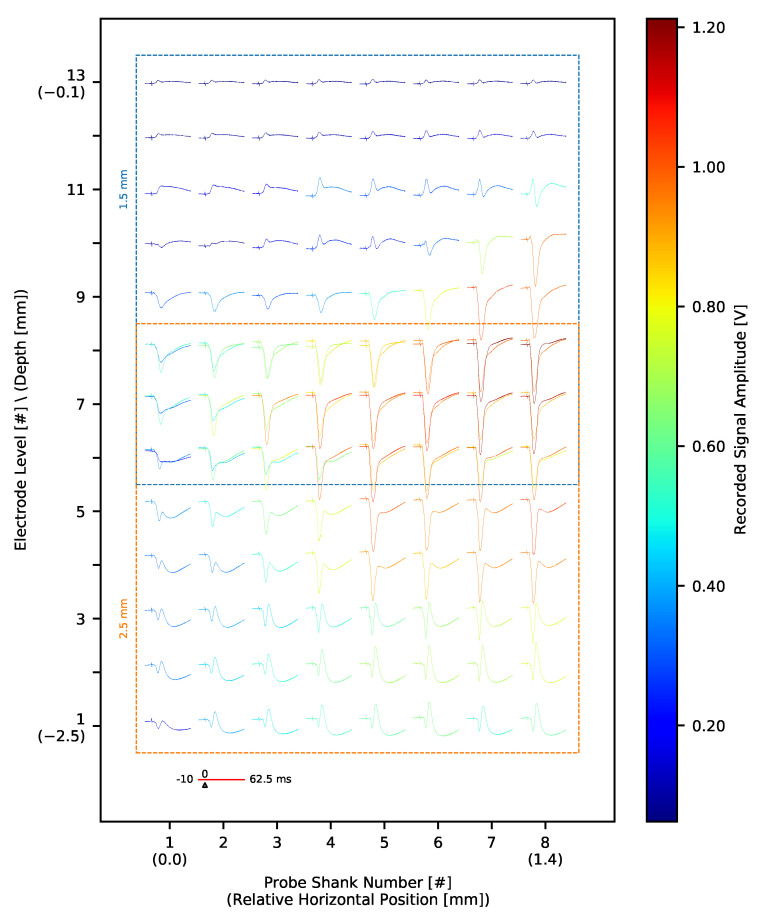
Average (n=100) evoked potential responses to whisker stimulation calculated for all MEA sites placed in a rat barrel cortex. To monitor activity of all cortical layers, the recording was repeated with MEA placed at the depth of 1.5 mm and 2.5 mm from the cortical surface. We can see that different MEA sites were effectively recording very similar signals from the same locations (marked with overlapping rectangles). Each average response is 62.5 ms long with 10 ms pre-trigger LFP and its amplitude is colour-coded (note that gain correction was not applied).

The application of sensory stimuli evokes neuronal activity in consecutive stages of the sensory pathway. Here we recorded typical responses evoked by whisker movements in the thalamus (Figure 26, compare [65,66]) and somatosensory cortex (Figure 27) after whiskers stimulation. EPs in somatosensory cortex showed characteristic depth-dependent profile, consistent with previously published work [67]. Both in the thalamus and the cortex, the largest EP amplitudes are observable in the areas of neuronal representation of stimuli. As confirmed also with post-mortem analysis of brain slices, in the thalamus the top-left corner (as for the Figure 26) of the electrode grid was best aligned with whisker representation; in the cortex, probe shanks 1 and 2 were outside the barrel field. The fact that the amplitude of evoked potentials in the thalamus is an order of magnitude smaller than in the cortex is typical. It results from the different cellular composition of these structures. The thalamus is built of small granular neurons, while cortical large pyramidal cells form strong electric dipoles and are responsible for high amplitude LFP waves.

The silicon probes used in this experiment allow for simultaneous recording of signals from a 1.4 mm × 1.4 mm block of tissue. In order to acquire signals across the full depth of a rat cortex (approximately 2 mm [68]), we performed recording in two steps (Figure 27). First, signals were recorded from upper cortical layers. In the next step, the probe was inserted deeper into the brain (by 1 mm) to acquire signals from deeper layers. Ultimately, signals from intermediate layers of tissue were recorded twice. As expected, the shapes of LFP waveforms recorded twice from the same locations are very similar. Slight differences are unavoidable since the evoked responses are very variable [69,70]. It is also difficult to exactly match electrode positions when moving MEA to a deeper level due to tissue viscosity.

#### 3.3.2. Electrical Stimulation

**Figure 28:**
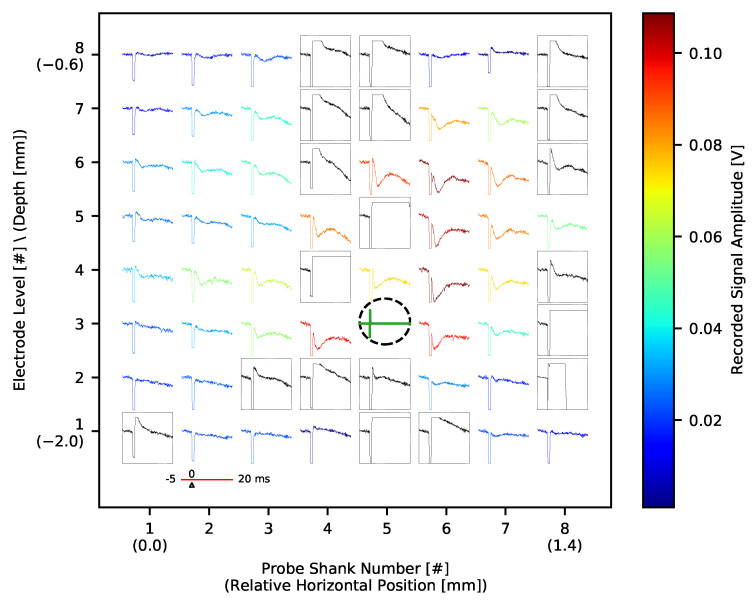
Average (n=10) evoked potential responses to current stimulation with charge-balanced bipolar pulse of amplitude 5.6 μA for all MEA sites placed in a rat barrel cortex (the deepest electrodes were placed 2 mm below cortical surface). The stimulating site was marked with a circle—the signal inside is the representation of the applied stimulation pulse (the timescale is the same as recorded signals, the amplitude scale is arbitrary). We can see that LFP response starts immediately after the end of stimulus and reaches the maximum after ∼5 ms. Several MEA sites exhibited strong artefacts shortly after stimulation pulse application (i.e., post-stimulus level 20 mV above the mean signal value before the pulse)—these were marked with rectangles and waveforms inside were clamped. Each average response is 25 ms long with 5 ms pre-trigger LFP and its amplitude is colour-coded except for sites with stimulation artefacts (note that gain correction was not applied).

We used electrical micro-stimulation with a plan to activate small populations of neurons around selected thalamic and cortical electrodes (Figure 15) and analyse the interaction of local networks and cortico-thalamic connections separately from layer 5 and layer 6. Bipolar electric pulses of increasing amplitude were generated with Neurostim-3 ASICs without stopping signal acquisition. The lowest amplitude currents (0.7 μA–1.0 μA) had no effect on neuronal activity. In the cortex, stronger impulses (1.4 μA and above) evoked responses recorded by neighbouring cortical electrodes as a small amplitude LFP waves (Electric Evoked Potentials (EEPs), Figure 28).

The trace of an electrical impulse can be seen on all the channels as a quick, square wave at a time zero. However, in some of the channels the signal was deformed also after the stimulus ended (black waveforms inside rectangles). If after the end of the stimulation pulse the value of the recorded signal was 20 mV above the mean signal value before the pulse, then such channel was marked as the one with an artefact. As a result, the almost entire MEA shank and several neighbouring electrodes had to be excluded from analysis after each stimulation. Since electrical stimulation was tested in one rat, no valid conclusions about artefact sources can be made. It might originate from the output current pulse shape, be the property of that specific MEA used, (e.g., the pattern of printed electric traces may be prone to interferences) or more probably be the convolution of those.

EEPs extracted from the data were the most visible for stimulation taking place at the 3rd MEA’s level, which corresponds to lower layer 5, which is composed of large pyramidal cells and axonal fibres passing from the thalamus to upper layers. When present, EEP was characterised by a negative wave starting immediately after current pulse application and lasting around 10 ms. The amplitude (colour-coded in Figure 28) scales with the output stimulation current amplitude (with response threshold $I_{stim_{Th}}\approx$ 1.4 μA) and exhibits a well-defined spatial profile indicating the centre of the activity. These parameters indicate that we observed weak depolarisation of local cells that have not reached the spike triggering threshold.

## 4. Conclusions and Plans for the Future

In this paper, we showed and discussed all components of the novel neural DAQ system for the Neurostim-3 ASIC, meant to work with up to 512 individual channels (each Neurostim-3 ASIC provides 64 of them) able to output current patterns and record incoming signals through the connected MEA. The hardware is managed by dedicated software, which provides efficient means of control of experiments, ASIC behaviour simulation and customisable real-time data visualisation. The latter can be performed locally or remotely since recorded data is distributed using either TCP or UDP communication. Although the software was written exclusively using *Python* programming language, it works perfectly on the middle-class computers and allows stimulation with up to 20,000 pulses per second without the requirement of *Output Bitstream* precomputation prior to the experiment.

The same software can be used to conduct hardware tests and actual experiments on the living brains. We showed that regardless of ASIC gain settings and input signal, its amplification factor remains stable. So as the channel analogue signal response, where the response distribution to the stimuli of the same input amplitude is well-defined. However, the actual gain varies between ASIC channels and tends to be higher for middle channels, which can be the property of the examined ASIC and may originate from the mismatch. On the other hand, knowing real per-channel gain of the given Neurostim-3 ASIC copy i.e., its calibration data, recorded waveforms can be easily scaled to obtain real input signal, which is crucial for offline data analysis. The bandwidth of each channel starts from ∼0.6 Hz which makes it suitable for most of the LFP measurements.

Besides, the ability to reconstruct complex pulses with the stimulation circuitry was checked and exhibited good agreement with the reference waveform. This allows us to think about the usage of sophisticated stimulation patterns in future experiments, possibly with stimulation artefact suppression with correction pulses, as described in [59]. This idea can take advantage of dynamic ASIC configuration, which would enable independent handling of connected ASICs and variable data sampling rate. The latter would be possible since the bit width of the *Real-Time Data Command* (and thus *Analogue Multiplexer* data sampling rate) can be varied according to the total number of enabled ASIC channels, eventually increasing channel signal sampling rate and current stimulation precision by an order of magnitude. Furthermore, it would allow more detailed measurement of the band-pass filter bandwidth. Enabling dynamic ASIC configuration, however, requires thorough rebuild of the system—starting from *Stimulation Stream Builder*, through *Demultiplexer*, up to *Visualisation Client*. This task is rather complex and poses threats of breaking communication mechanisms with ASICs which can be extremely difficult to track and repair, but the effort would pay off with flexibility of the definition of future experiments’ protocols.

The DAQ was also tested in the experiments with living rats’ brains. We measured spontaneous activity and EPs in the thalamus and barrel cortex and observed a broad range of LFPs and spiking activity, proving that we obtained physiological signals. What is more, when either whisker or electrical stimuli were applied, the results exhibit depth-dependent profiles near the areas of neuronal representation of stimuli. However, in the latter case, tissue response was less prevalent and contaminated by stimulation artefacts. Although, these effects require more investigation, at this point we are confident that presented DAQ is capable of conducting neurophysiological experiments with animals. One way of electric stimulation efficiency improvement would be to check whether simultaneous stimulation from clusters of neighbouring MEA’s sites would evoke clean spiking activity. Also, selecting MEA with larger electrodes would allow us to use currents of increased amplitude up to the maximum available 15 μA, since at the moment, we are constrained by the current density so that not to evaporate the material of an electrode.

As for the hardware side, currently available IB can handle up to two ABs with single Neurostim-3 ASIC on them, enabling carrying out experiments with two MEAs with maximum of 128 (2×64) independent input/output channels. Our DAQ system, however, can be scaled easily by:

- Multiplication of internal circuitry on the IB responsible for the management of additional ASICs (up to eight).
- Building ABs with two Neurostim-3 ASICs on them.

These enhancements will not only allow conducting measurements with more channels but also give the opportunity of usage of denser MEAs. The presented software is already prepared to handle that since the entire data processing pipeline assumes that eight Neurostim-3 ASICs are connected and working. Hence, we are planning to use four 128 -channel MEAs in the forthcoming experiments making use of the system’s designed capacity (new IB and ABs are currently being implemented). Moreover, the next revision of Neurostim-3 ASIC was developed, and it is going to be characterised as well.

To conclude, the whole system is ready to be used by neurobiologists, today. The plans of making stimulation protocol more flexible with variable-rate data sampling will result in improved versatility of the Neurostim-3 DAQ and lead to living brain activity measurements of better quality.

## Figures and Tables

**Table 1:** List of software dependencies.

Purpose	Tool Name	Version	References
Programming language	*CPython*	3.8.3 64bit	[31]
Graphical User Interface (GUI)	*PyQt5*	5.14	[45]
Plotting tool	*PyQtGraph*	0.11	[44]
Data storage	*HDF5*	1.8.14 64bit	[40]
	*h5py*	1.18	[47]
External device communication	*NI-DAQmx*	18.1f1	[20,21]
	*NI-VISA*	18.5	[48,49]
	*PyVISA*	1.10	[50]
Shared memory implementation	*arrayqueues*	1.2.0b0	[51]
Numerical data processing	*NumPy*	1.18	[39]
	*SciPy*	1.4.1	[52]
Python to machine code compiler	*Numba*	0.49.1	[38]
Process monitor	*psutil*	5.7	[53]

**Table 2:** Specified gain and *DAC 10bit* pairs used during channel gain characteristics measurement.

Gain $\left[V/V\right]$	100	150	200	250	300	400	500
*DAC 10bit*	120	80	60	48	40	30	24

**Table 3:** Performance metrics of the *Stimulation Stream Builder* during high-rate pulse insertion stress test.

	Scheduling Configuration			
	Normal	Affinity	Priority	Affinity + Priority
Mean time per *Output Bitstream* chunk [ms]	474	450	441	442
σ [ms]	15.3	7.5	4.2	4.7
Mean Central Processing Unit (CPU)	97	100	92	100
core utilization [%]				

## Data Availability

Data available on request. The data presented in this study are available on request from the corresponding author.

## Abbreviations

The following abbreviations are used in this manuscript:

***µ*****LED**	micro Light Emitting Diode. 2
**2-D**	two-dimensional. 11, 15
**4K**	UHD 4K Ultra High Definition. 15
**AB**	ASIC Board. 4–7, 19, 23, 35
**ADC**	Analogue-Digital Converter. 56, 11
**ASIC**	Application Specific Integrated Circuit. 1–13, 15–24, 26, 27, 29, 30, 32–35
**CMOS**	Complementary Metal–Oxide–Semiconductor. 5–7
**CPU**	Central Processing Unit. 8, 15, 19, 20, 24–26
**DAC**	Digital-Analogue Converter. 18,20
**DAQ**	Data Acquisition. 1–3, 15, 16, 19, 20, 22, 25, 26, 31, 34–36
**DLL**	Dynamic-Link Library. 11
**DSP**	Digital Signal Processing. 15
**EEG**	Electroencephalography. 1
**EEP**	Electric Evoked Potential. 33, 34
**EP**	Evoked Potential. 23, 31, 35
**FFT**	Fast Fourier Transform. 15
**FSM**	Finite State Machine. 9, 16
**GIL**	Global Interpreter Lock. 8
**GPIB**	General Purpose Interface Bus. 5, 9
**GUI**	Graphical User Interface. 15, 17, 18
**GZIP**	GZIP. 13, 16
**HDF5**	Hierarchical Data Format. 13
**HT**	Hyper-Threading. 20
**i.p.**	Intraperitoneal Injection. 23
**IB**	Interface Board. 4–7, 11, 17, 23, 35
**JSON**	JavaScript Object Notation. 10
**LDO**	Low-Dropout Regulator. 7
**LFP**	Local Field Potential. 1, 2, 15, 21, 23, 29, 31–35
**LVDS**	Low-Voltage Differential Signalling. 5, 6
**MEA**	Multi-Electrode Array. 1–6, 17, 19, 23, 24, 31–35
**MEG**	Magnetoencephalography. 1
**NIC**	Network Interface Card. 20, 26
**NS3**	Neurostim-3. 4
**PBS**	Phosphate Buffered Saline. 23
**PC**	Personal Computer. 1, 3, 26
**PEG**	Polyethylene Glycol. 23
**PSD**	Power Spectral Density. 21, 29
**PSU**	Power Supply Unit. 5, 7, 9
**RAM**	Random Access Memory. 20, 24
**SNR**	Signal-To-Noise Ratio. 28, 31
**TCP**	Transmission Control Protocol. 7, 8, 11, 13, 14, 26, 34
**UDP**	User Datagram Protocol. 7, 8, 14, 34
